# Lewy pathology of the esophagus correlates with the progression of Lewy body disease: a Japanese cohort study of autopsy cases

**DOI:** 10.1007/s00401-020-02233-8

**Published:** 2020-11-05

**Authors:** Zen-ichi Tanei, Yuko Saito, Shinji Ito, Tomoyasu Matsubara, Atsuko Motoda, Mikihiro Yamazaki, Yasuhiro Sakashita, Ito Kawakami, Masako Ikemura, Shinya Tanaka, Renpei Sengoku, Tomio Arai, Shigeo Murayama

**Affiliations:** 1 Department of Neurology and Neuropathology (the Brain Bank for Aging Research), Tokyo Metropolitan Geriatric Hospital and Institute of Gerontology, 35-2 Sakae-cho, Itabashi-ku, Tokyo, 173-0015 Japan; 2 Department of Cancer Pathology, Faculty of Medicine, Hokkaido University, Sapporo, Japan; 3 Department of Pathology, Toranomon Hospital, Tokyo, Japan; 4 Department of Clinical Neuroscience and Therapeutics, Hiroshima University Graduate School of Biomedical and Health Sciences, Hiroshima, Japan; 5 Department of Neurology, The Jikei University School of Medicine, Tokyo, Japan; 6 Department of Neurology and Neurobiology of Aging, Kanazawa University Graduate School of Medical Sciences, Kanazawa, Japan; 7 Dementia Research Project, Tokyo Metropolitan Institute of Medical Science, Tokyo, Japan; 8 Department of Pathology, Graduate School of Medicine, The University of Tokyo, Tokyo, Japan; 9 Institute for Chemical Reaction Design and Discovery (WPI-ICReDD), Hokkaido University, Sapporo, Japan; 10 Department of Pathology, Tokyo Metropolitan Geriatric Hospital and Institute of Gerontology, Tokyo, Japan; 11 Brain Bank for Neurodevelopmental, Neurological and Psychiatric Disorders, United Graduate School of Child Development, Osaka University, Osaka, Japan

**Keywords:** Lewy body disease, Parkinson’s disease, α-Synuclein, Esophagus, Enteric nervous system, Peripheral nervous system

## Abstract

Lewy body disease (LBD) is a spectrum of progressive neurodegenerative disorders characterized by the wide distribution of Lewy bodies and neurites in the central and peripheral nervous system (CNS, PNS). Clinical diagnoses include Parkinson’s disease (PD), dementia with Lewy bodies, or pure autonomic failure. All types of LBD are accompanied by non-motor symptoms (NMSs) including gastrointestinal dysfunctions such as constipation. Its relationship to Lewy body-related α-synucleinopathy (Lewy pathology) of the enteric nervous system (ENS) is attracting attention because it can precede the motor symptoms. To clarify the role of ENS Lewy pathology in disease progression, we performed a clinicopathological study using the Brain Bank for Aging Research in Japan. Five-hundred and eighteen cases were enrolled in the study. Lewy pathology of the CNS and PNS, including the lower esophagus as a representative of the ENS, was examined via autopsy findings. Results showed that one-third of older people (178 cases, 34%) exhibited Lewy pathology, of which 78 cases (43.8%) exhibited the pathology in the esophagus. In the esophageal wall, Auerbach’s plexus (41.6%) was most susceptible to the pathology, followed by the adventitia (33.1%) and Meissner’s plexus (14.6%). Lewy pathology of the esophagus was significantly associated with autonomic failures such as constipation (*p* < 0.0001) and among PNS regions, correlated the most with LBD progression (*r* = 0.95, *p* < 0.05). These findings suggest that the propagation of esophageal Lewy pathology is a predictive factor of LBD.

**Electronic supplementary material:**

The online version of this article (10.1007/s00401-020-02233-8) contains supplementary material, which is available to authorized users.

## Introduction

Lewy body disease (LBD) is a neurodegenerative disorder in which Lewy bodies (LBs) and neurites appear in the central and peripheral nervous systems (CNS, PNS). It is clinically diagnosed as Parkinson’s disease (PD), dementia with Lewy bodies (DLB), or pure autonomic failure (PAF) [36, 44–47, 51–53, 86]. The main component of LBs has proven to be phosphorylated α-synuclein [3, 28, 79], thus allowing Lewy pathology—the accumulation and distribution of phosphorylated α-synuclein—to be examined in the CNS and PNS of people with LBD [4, 42, 70, 71].

In addition to well-known motor symptoms, patients with PD might also exhibit non-motor symptoms (NMSs) such as autonomic failure, psychological symptoms such as depression, apathy, and psychosis, cognitive decline, or rapid eye movement sleep-behavior disorder [65]. Some NMSs have been shown to precede the motor symptoms or clinical diagnosis of PD [1, 34, 43, 60, 66, 75]. Indeed, gastrointestinal dysfunction—a primary NMS in PD—has recently been attracting attention because Lewy pathology in the enteric nervous system (ENS) can be a predictive marker for PD/DLB [40, 80, 81, 87].

The way that Lewy pathology propagates was first advanced by Braak and colleagues [11, 12]. The pathology of the PNS is noteworthy in that α-synuclein accumulation can be seen in numerous organs and tissues during the pre-symptomatic or symptomatic phase [4, 21, 88], including sympathetic ganglia [20, 26, 83], the heart [27, 41, 56, 59, 61–63], adrenal gland [20, 29], skin [22, 23, 25, 39, 58, 78, 91], olfactory mucosa [30], olfactory epithelium [73], olfactory bulb [6, 11, 16, 19, 38, 64, 76], posterior pituitary gland [37], spinal cord [14], dorsal root ganglia [82], submandibular gland [18], upper aerodigestive tract [57], gallbladder [40], and genitourinary tract [4]. Lewy pathology of the ENS was first reported in patients who had PD with dysphagia [68] and then in those who had PD with megacolon [48]. Subsequently, LBs have been shown to be most frequent in the lower esophagus [4, 31, 89, 90]. Autopsy studies have estimated the frequency of α-synuclein deposition in the ENS to be between 50–100% in PD/DLB, 14–100% in incidental LBD, and 0–52% in controls [2, 4, 5, 7, 10, 17, 31, 33, 57]. Further, analyses of surgical specimens or biopsies of the ENS have revealed this pathology up to 20 years before the onset of the disease with a positive rate of 13–100% [40, 74, 77, 80, 81]. Therefore, the ENS is vulnerable to Lewy pathology in LBD. However, the precise prevalence of ENS pathology in older people or in those who have pre-symptomatic LBD, and the specificity during LBD development, remain unclear.

To clarify the role of Lewy pathology of the ENS in the LBD progression, we performed a clinicopathological study using community-based, autopsy-confirmed cohorts of older Japanese people from the Brain Bank for Aging Research (BBAR).

## Materials and methods

### Tissue source

Tissue samples were obtained from autopsy cases that took place at the Tokyo Metropolitan Geriatric Hospital and Institute of Gerontology (TMGHIG). TMGHIG is located in urban Tokyo and provides community-based general and emergency services to the older (+ 65 years) population, including patients with dementia or neurodegenerative diseases. TMGHIG performed 738 autopsies between October 2008 and June 2018 and obtained consent to register them to the BBAR, which is approved by the institutional ethics review committee. Among these cases, 202 excluded from this study because they lacked consent for craniotomy. Among the remaining 536 cases available for this study, we excluded 8 that had undergone brain autopsy (7 cases of Creutzfeldt–Jakob disease and one case of PD), 6 that exhibited multiple system atrophy (another type of α-synucleinopathy), and 4 that showed total brain necrosis (immunocytochemistry not applicable). This left 518 cases for analysis in this study.

### Histology

We examined the brain, spinal cord, olfactory bulb and tract, and the PNS, including the sympathetic ganglia [82], anterior wall of the left ventricle of the heart [56], lower esophagus (as a representative of the ENS) [4, 31, 89, 90], adrenal gland [29], and skin of the upper arm and thigh [39]. The CNS was examined as previously reported [30, 70, 71, 76]. Briefly, the cerebral hemisphere, cerebellum, and brain stem were first dissected in the sagittal plane during the autopsy to separate the hemispheres: one to be frozen and the other for fixation. The frozen side of the brain was then cut in several 8-mm slices: cerebral hemisphere in the coronal plane, cerebellum in the sagittal plane, and brain stem in the horizontal plane. The PNS was examined as follows. Six sympathetic ganglia at the level of the heart and adrenal glands with periadrenal adipose tissue were sliced in the maximum sections. Transmural myocardial tissue was obtained from the anterior wall of the left ventricle of the heart. The lower esophagus and skin were dissected in a 2-cm length with the mucosa to adventitia or the epidermis to subcutaneous fat tissue in two sections. The PNS and some parts of the frozen brain sample, including the frontal and temporal pole, parietal lobe (intraparietal sulcus), primary visual cortex, posterior hippocampus, amygdala, cerebellum including the dentate nucleus, substantia nigra, and the olfactory bulb were dissected in a block less than 2 cm × 1.5 cm before rapid freezing and fixed with 4% paraformaldehyde (PFA) for 48 h. The remaining parts of the frozen sample were preserved at − 80 °C for further biochemical and molecular analyses. The other half of the brain was fixed with 20% buffered formalin (WAKO, Japan) for 7–13 days and cut in 5-mm slices as described above. The representative areas were embedded in paraffin and 6-μm-thick sections were prepared for hematoxylin and eosin staining (H&E), the Klüver-Barrera method, and immunohistochemistry. Further, selected slides were stained with Gallyas-Braak and modified methenamine-silver staining for evaluation of changes related to senility.

### Immunohistochemistry and evaluation

Sections were immunostained with Ventana BenchMark GX autostainer (Roche, Switzerland) and iView DAB Detection Kit (Roche, Switzerland) according to the manufacturers’ instructions. The antibodies used in this study were mouse monoclonal anti-phosphorylated-α-synuclein antibody (pSyn#64, 1:20,000, a gift from Dr. Iwatsubo [70]; now available for purchase from FUJIFILM Wako Pure Chemical Corporation, Japan), mouse monoclonal anti-amyloid-β antibody (clone 12B2, 1:50, IBL, Japan), rabbit monoclonal anti-phosphorylated-tau antibody (clone AT8, 1:1000, Fujirebio, Japan), and mouse monoclonal anti-phosphorylated-TAR DNA-binding protein 43 (TDP-43) antibody (pS409/410, 1:10,000, a gift from Dr. Hasegawa; now available for purchase from Cosmo Bio Co Ltd, Japan [85]). Two anti-phosphorylated-α-synuclein antibodies—rabbit polyclonal antibody (PSer129, 1:100, a gift from Dr. Iwatsubo [28] and Dr. Akiyama [4]) and rabbit monoclonal antibody (MJF-R13, ab168381, 1:80,000, Abcam, UK)—and anti-non-phosphorylated-α-synuclein antibody (LB509, 1:100, gift from Dr. Iwatsubo [3]; now available for purchase as ab27766, Abcam, UK or as SIG-39725, Biolegend, USA) were employed to confirm positive immunoreactivity in some select cases after protease treatment (Supplementary Fig. 1, online resource).

Histological and immunohistochemical evaluations were performed via light microscopic observation (Eclipse Ni, Nikon, Japan). For clinicopathological staging of the Lewy pathology, we evaluated the BBAR LB stage (Table 1; modified from Saito et al. 2003 [70] and Funabe et al. 2013 [30]), DLB Consensus Guidelines [51–53], and Braak LB stage [11, 12]. Semi-quantitative analyses for the CNS and PNS Lewy pathology were performed using the grading system from the third report of the DLB consortium [52]. Lewy pathology distribution in the esophageal wall was analyzed with the following subdivisions: mucosa, muscularis mucosa, submucosa including Meissner’s plexus, muscularis propria including Auerbach’s plexus, and adventitia.

**Table 1:** BBAR LB stage^a^

Stage	pSyn-IR	LB	Loss of pigmentation of the LC/SN	Parkinsonism	Dementia^b^	LB score^c^	Diagnosis
0	−	−	−	−	−	0	
0.5	+	−	−	−	−	0	Earliest LBD
1	+	+	−	−	−	0–10	Preclinical LBD
2	+	+	+	−	−	0–10	Prodromal LBD
3	+	+	+	+	−	0–10	PD
4	+	+	+	+ / −	+	3–6	PDD/DLBT
5	+	+	+	+ / −	+	7–10	PDD/DLBN

*BBAR* brain bank for aging research, *pSyn-IR* phosphorylated α-synuclein immunoreactivity, *LB* Lewy body, *LC* locus coeruleus, *SN* substantia nigra, *LBD* Lewy body disease, *PD* Parkinson’s disease, *PDD* Parkinson’s disease with dementia, *DLBT* dementia with Lewy bodies, transitional form, *DLBN* dementia with Lewy bodies, neocortical form; +  presence; −  absence

^a^Ref. Funabe S et al. [30]

^b^NIA-AA criteria [54]

^c^DLB consensus guideline 1996 [53]

For staging of amyloid β and phosphorylated tau, we evaluated the Braak senile plaque stage [9], CERAD score [55], Thal senile plaque phase [84], Braak neurofibrillary tangle stage [8, 9], and Saito argyrophilic grain stage [72]. For pTDP-43, we scored the medial temporal lobe (amygdala and anterior hippocampus), medulla oblongata, and lumbar spinal cord as previously reported [85].

### Clinicopathological and genetic information

Information including age, sex, presence or absence of autonomic failures (such as severe constipation, orthostatic hypotension, or urinary dysfunction), Parkinsonism, and dementia was obtained from the BBAR database and extracted from medical records by neurologists. The Mini-Mental State Examination, the Revised Hasegawa’s dementia scale, and the Clinical Dementia Rating were used for evaluating dementia. The interval between the last assessment upon admission and time to death was less than 2 months in most of the individuals. Brain weights, neuropathological diagnoses, and apolipoprotein (*APOE*) status were based on the BBAR database and reviewed by pathologists.

### Statistical analysis

Quantitative and semi-quantitative data were statistically analyzed by the χ^2^ test, Student’s *t* test, Mann–Whitney *U* test, and Spearman's rank correlation coefficient using GraphPad Prism 6 (GraphPad Software, San Diego, CA).

## Results

### Prevalence of Lewy pathology in older individuals

**Fig. 1:**
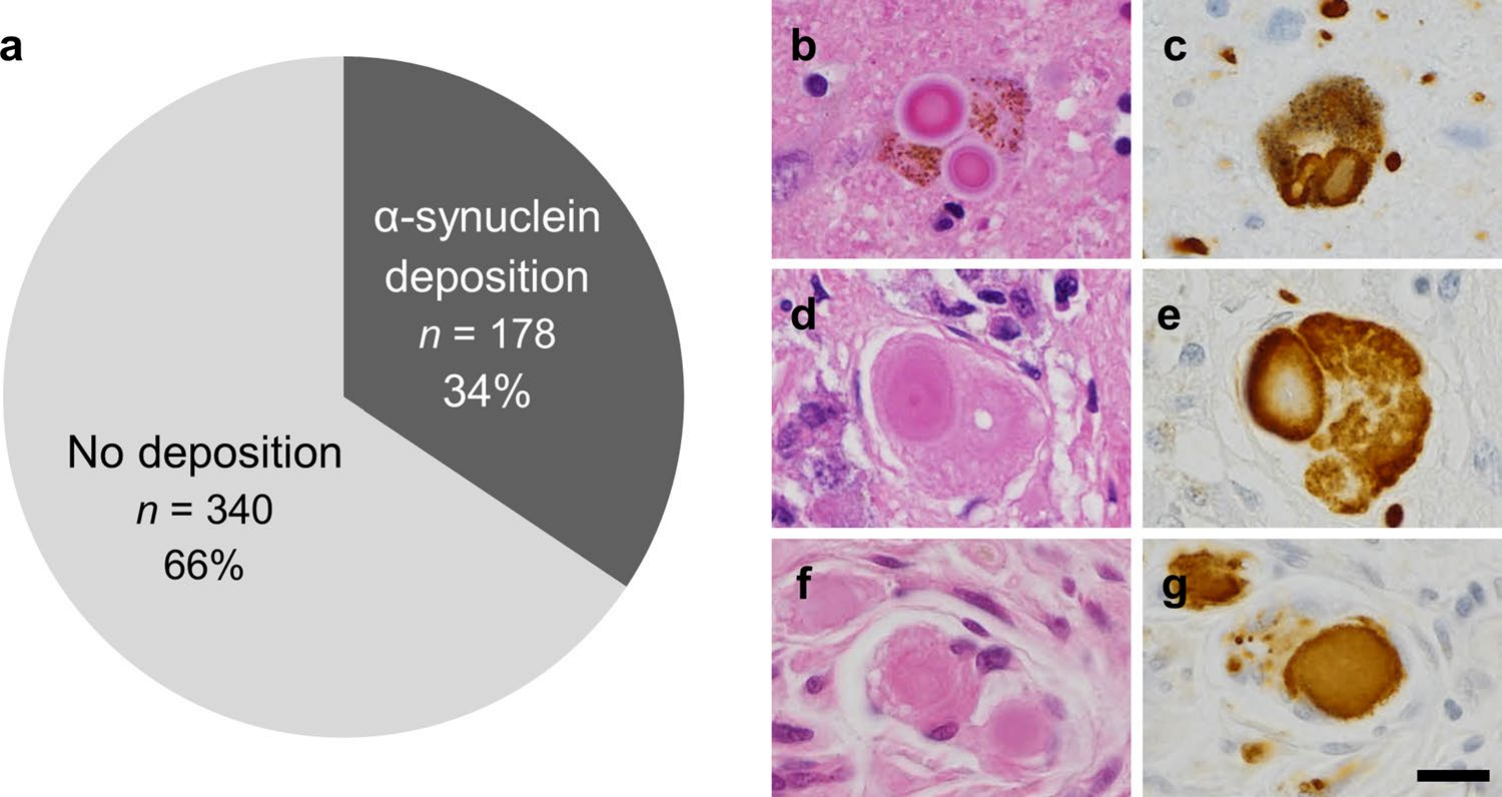
Prevalence of Lewy pathology in the 518 BBAR cases. **a** One-third of older individuals exhibited α-synuclein accumulation. **b**–**g** LB in the locus coeruleus (**b**, **c**), adrenal gland (**d**, **e**), and esophagus (**f**, **g**). H&E (**b**, **d**, **f**) and pSyn#64 (**c**, **e**, **g**). Scale bar = 10 μm. *BBAR* the Brain Bank for Aging Research

Among the 518 autopsy cases, 73% of which died from respiratory diseases, malignant neoplasm, or cardiovascular disease (Supplementary Fig. 2, Supplementary Table 1, online resource), approximately one-third of older individuals exhibited Lewy pathology either in the CNS and/or the PNS (178/518: 34%, Fig. 1; Supplementary Fig. 3, online resource). In most of these cases, the pathology was observed in both the CNS and PNS (121/178: 68%). PNS-only pathology was found in 9 cases (5%), including 5 cases in which it was limited to the sympathetic ganglia (Supplementary Table 3, online resource). CNS-only pathology was observed in 48 cases (27%), of which 23 were amygdala-predominant, 14 were brainstem-predominant, 8 were limbic, and 3 were olfactory-bulb-only according to the LB type pathology by DLB Consensus Guidelines [51–53]. Fifteen cases were neuropathologically diagnosed as Alzheimer’s disease (AD). None exhibited Lewy pathology in the esophagus only or esophageal α-synuclein accumulation without CNS pathology.

The clinicopathological characteristics are summarized in Table 2. The positivity of Lewy pathology was significantly associated with age (male: positive cases, mean age at death = 81.1 ± 9.5 years; negative cases, 77.9 ± 11.8 years; female: positive cases, 86.4 ± 8.5 years; negative cases, 82.3 ± 11.0 years; *p* < 0.05) and higher pathological stages of neurofibrillary tangles (*p* < 0.0001) or senile plaques (*p* < 0.05), but not with sex, *APOE* status, or stages of argyrophilic grains or pTDP-43 (data not shown).

**Table 2:** Characteristics of the 518 BBAR cases

	Lewy pathology		*P* value
	Positive	Negative	
Clinical and genetic characteristics			
Gender (*n*)			
Male	98 (18.9%)	205 (39.6%)	0.26
Female	80 (15.4%)	135 (26.1%)	
Mean Age at death (*y*) (SD, range)			
Male	81.1 (9.5, 49–99)	77.9 (11.8, 25–99)	0.04
Female	86.4 (8.5, 61–104)	82.3 (11.0, 24–111)	0.004
* APOE* (*n*)			
ε2/ε3	11 (2.1%)	32 (6.2%)	0.7143
ε3/ε3	130 (25.1%)	224 (43.2%)	
ε2/ε4	1 (0.2%)	2 (0.4%)	
ε3/ε4	31 (6.0%)	58 (11.2%)	
ε4/ε4	3 (0.6%)	5 (1.0%)	
Neuropathological factors			
Brain weight (*g*, mean)	1208	1234	0.051
(SD, range)	(132, 758–1666)	(145, 730–1937)	
Braak NFT stage (*n*)			
0–II	92 (17.8%)	249 (48.1%)	< 0.0001
III–VI	85 (16.4%)	91 (17.6%)	
Braak SP stage (*n*)			
0–A	109 (21.0%)	257 (49.6%)	0.0011
B–C	68 (13.1%)	83 (16.0%)	
CERAD score (*n*)			
0–A	91 (17.6%)	237 (45.8%)	0.0008
B–C	77 (14.9%)	103 (19.9%)	
Thal phase (mean)	2.5	2.1	0.0021

*BBAR* the Brain Bank for Aging Research, *CERAD* the Consortium to establish a registry for Alzheimer’s disease, *NFT* neurofibrillary tangle, *SD* standard deviation, *SP* senile plaque

### Lewy pathology of the PNS

To clarify the specificity of esophageal Lewy pathology in the PNS, we compared the positivity in the esophagus, sympathetic ganglia, heart, adrenal gland, and skin with the BBAR LB stage.

**Fig. 2:**
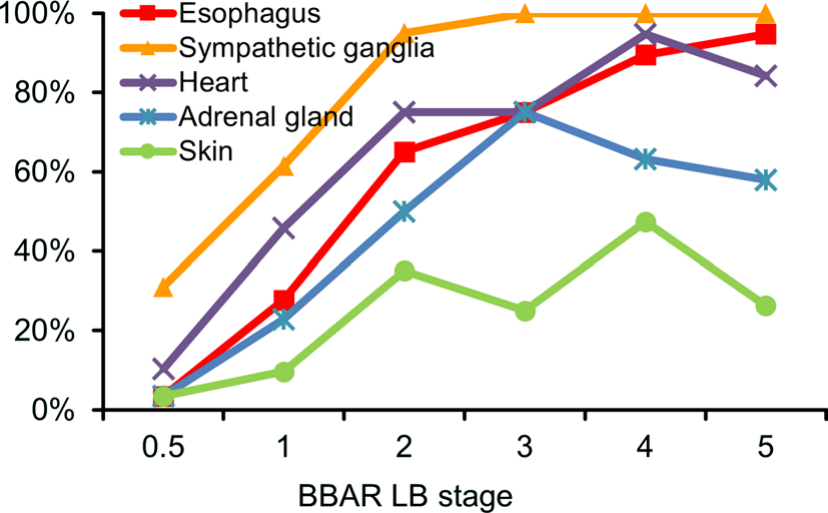
Lewy pathology of the PNS at different BBAR LB stages. The positive rate of Lewy PNS pathology increased with the BBAR LB stage. Among PNS regions, the correlation with the BBAR LB stage was highest in the esophagus (*r* = 0.95), followed by the sympathetic ganglia (*r* = 0.85), heart (*r* = 0.87), adrenal gland (*r* = 0.81), and skin (*r* = 0.71; Spearman’s rank correlation coefficient, all *p* < 0.05). Positivity in the esophagus gradually increased with stage, while that in the heart and adrenal gland decreased at the last stage. The sympathetic ganglia exhibited the highest rate of pathology among the regions in the PNS. Details are summarized in Supplementary Table 2, online resource. *BBAR* the Brain Bank for Aging Research, *LB* Lewy body, *PNS* peripheral nervous system

Among 178 cases positive for Lewy pathology, the mean percentages of α-synuclein deposition were as follows: sympathetic ganglia, 70.2% (125 cases); heart, 55.1% (98); esophagus, 43.8% (78); adrenal gland, 33.7% (60); skin, 18.0% (32). The incidence increased with the progression of BBAR LB stage, with the percentages in the esophagus correlating the most (*r* = 0.95), followed by the sympathetic ganglia (*r* = 0.85), the heart (*r* = 0.87), adrenal gland (*r* = 0.81), and skin (*r* = 0.71, Spearman's rank correlation coefficient, all *p* < 0.05, Fig. 2; Supplementary Table 2, online resource). Although the percentage of α-synuclein in the esophagus was almost the same as that in the heart and adrenal gland, the esophageal positivity gradually increased and reached 94.7% (18/19) by stage 5. In contrast, the incidence in the heart and adrenal gland decreased with the stage of progression (heart, stage 4–5: 94.7% (18/19) to 84.2% (16/19); adrenal gland, stage 3–5: 75.0% (6/8) to 57.9% (11/19)). The sympathetic ganglia exhibited the highest incidence and Lewy pathology was observed in all cases that were at least stage 3.

### Lewy pathology of the esophagus and clinicopathological characteristics

To examine the characteristics of the esophageal Lewy pathology, we compared the presence or absence of the pathology with clinical, genetic, and neuropathological factors (Table 3). Analysis showed that the pathology was significantly associated with autonomic failures, Parkinsonism, and the stage of LB pathology as determined by the 4th DLB Consensus Guidelines, the BBAR LB stage [70], and the Braak LB stage (*p* < 0.0001) [11, 12], but not with age, sex, or brain weight. Lewy pathology tended to be associated with dementia, although the tendency did not reach statistical significance (*p* = 0.0562).

**Table 3:** Esophageal Lewy pathology and clinicopathological factors among 178 older people who showed Lewy pathology in their nervous systems

	Esophageal Lewy pathology			*P* value
	Positive		Negative	
Clinical and genetic characteristics				
Age at death (*y*, mean)	84.3		82.8	0.675
Gender (*n*)				
Male	40 (22.5%)		58 (32.6%)	0.4479
Female	38 (21.3%)		42 (23.6%)	
* APOE* (*n*)				
ε2/ε3	6 (3.4%)		5 (2.8%)	ND
ε3/ε3	55 (30.9%)		75 (42.1%)	
ε2/ε4	1 (0.6%)		0 (0%)	
ε3/ε4	13 (7.3%)		18 (10.1%)	
ε4/ε4	2 (1.1%)		1 (0.6%)	
Constipation (*n*)				
Positive	23 (12.9%)		5 (2.8%)	< 0.0001
Negative	55 (30.9%)		95 (53.4%)	
Other autonomic failures (*n*)				
Positive	18 (10.1%)		5 (2.8%)	0.0005
Negative	60 (33.7%)		95 (53.4%)	
Parkinsonism (*n*)				
Positive	31 (17.4%)		9 (5.1%)	< 0.0001
Negative	47 (26.4%)		91 (51.1%)	
Dementia (*n*)				
Positive	48 (27.0%)		46 (25.8%)	0.0562
Negative	30 (16.9%)		54 (30.3%)	
Neuropathological factors				
Brain weight (*g*, mean)	1212		1205	0.7406
DLB 4th consensus report (*n*)				
Diffuse neocortical	44 (24.7%)		5 (2.8%)	< 0.0001
Limbic	22 (12.4%)		23 (12.9%)	
Brainstem-predominant	11 (6.2%)		32 (18.0%)	
Amygdala-predominant	1 (0.6%)		28 (15.7%)	
BBAR LB stage (*n*)				
0–2	37 (20.8%)		95 (53.4%)	< 0.0001
3–5	41 (23.0%)		5 (2.8%)	
Braak LB stage (*n*)				
0–3	21 (11.8%)		85 (47.8%)	< 0.0001
4–6	57 (32.0%)		15 (8.4%)	

*BBAR* the Brain Bank for Aging Research, *DLB* dementia with Lewy bodies, *LB* Lewy body, *ND* not determined

### Lewy pathology in the esophageal wall

**Fig. 3:**
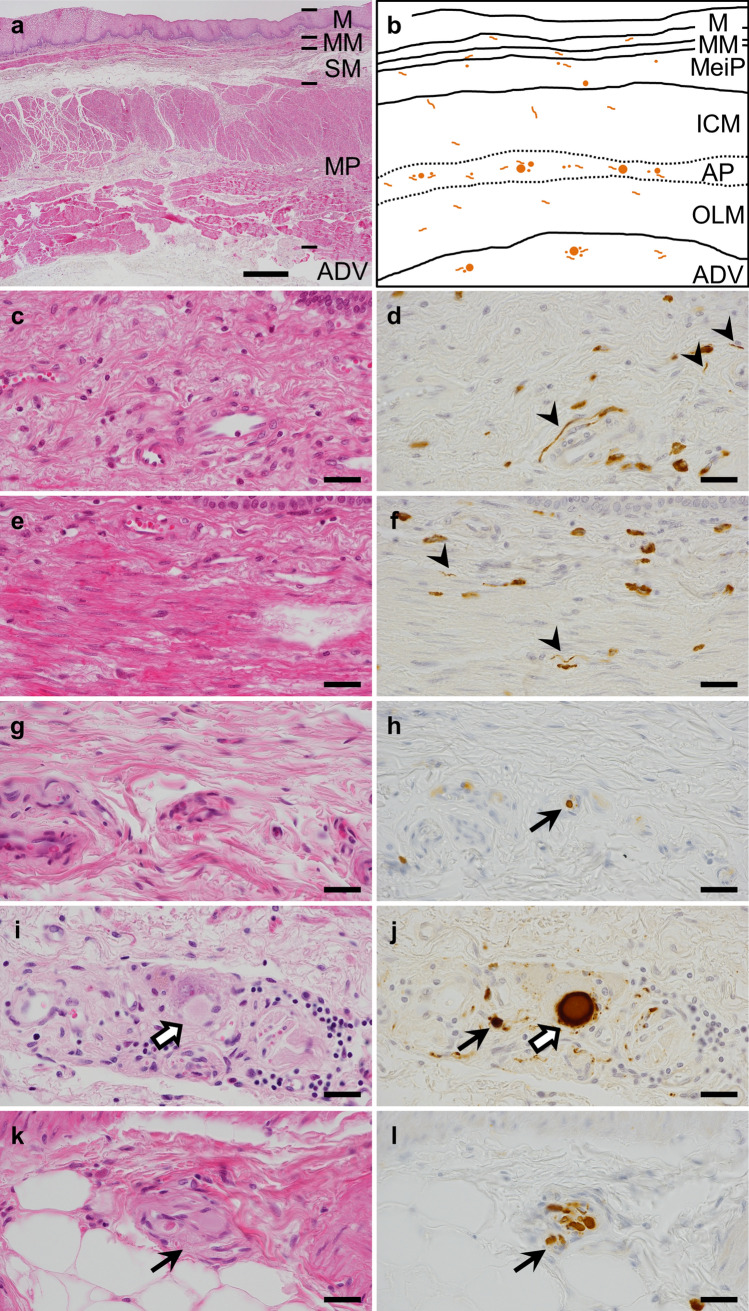
Lewy pathology in the esophageal wall. **a** Low magnification view of the esophageal wall and its anatomy. **b** Schema of Lewy pathology, shown in orange spherical or neuritic structures in the esophageal wall. **c**–**l** High magnification view of the mucosa, especially lamina propria (**c**, **d**), muscularis mucosa (**e**, **f**), submucosa (**g**, **h**), muscularis propria (**i**, **j**) and adventitia (**k**, **l**). LB were observed in Auerbach’s plexus in the MP (white arrows, **i**, **j**) and ADV. Lewy neurites or pSyn#64-immunoreactive (IR) aggregates were found in Meissner’s plexus of the SM, Auerbach’s plexus, and the ADV (arrows, **h**, **j**, **k**, **l**). Only pSyn#64-IR neurites were observed in the M and MM (arrowheads, **d**, **f**). Non-specific staining of stromal cell cytoplasm—most were mast cell granules—was observed in the background (**d**, **f**). H&E (**a**, **c**, **e**, **g**, **i**, **k**) and pSyn#64 (**d**, **f**, **h**, **j**, **l**). Scale bar = 500 µm (**a**), 20 µm (**c**–**l**). *LB* Lewy body, *M* mucosa, *MM* muscularis mucosa, *SM* submucosa, *MeiP* Meissner’s plexus, *MP* muscularis propria, *ICM* inner circular muscle layer of MP, *OLM* outer longitudinal muscle layer of MP, *AP* Auerbach’s plexus, *ADV* adventitia

To examine the locus of Lewy pathology in the esophageal wall, we semi-quantified the density of the α-synuclein accumulation in separate regions of the wall (Fig. 3a, b) and compared each density with the BBAR LB stage.

**Fig. 4:**
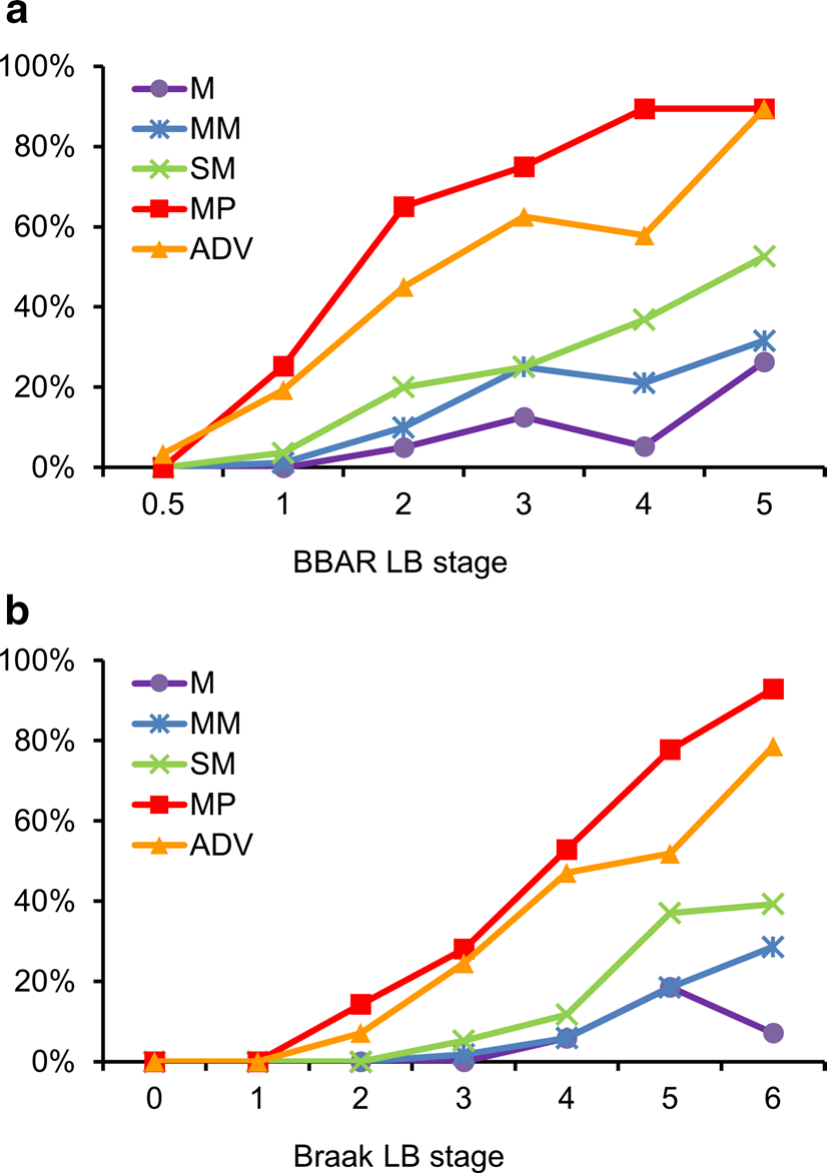
Lewy pathology in the esophageal wall at different BBAR and Braak LB stages. **a** BBAR LB stages; The positive rate of Lewy pathology increased with BBAR LB stage. The highest rates were found in the MP and ADV (mean rates of 41.6% and 33.1% respectively), reaching 89.5% at stage 5. Lewy pathology was observed less in the M and MM. **b** Braak LB stages; The positivity of Lewy pathology also increased with Braak LB stage. The highest rates were found in the MP, reaching 92.9% at stage 6. Lewy pathology was observed less in the M and MM. Details are summarized in Table 4a, b. *BBAR* the Brain Bank for Aging Research, *LB* Lewy body, *M* mucosa, *MM* muscularis mucosa, *SM* submucosa, *MP* muscularis propria, *ADV* adventitia

LBs were observed in Auerbach’s plexus and the adventitia (ADV) as homogenous and spherical cytoplasmic inclusions with clear halos via H&E and round inclusions via anti-α-synuclein immunohistochemistry (Fig. 3i, j). Additionally, Lewy neurites or pSyn#64-immunoreactive (IR) aggregates (round or elongated cytoplasmic or neuronal process inclusions) were found in Meissner’s plexus, Auerbach’s plexus, and the ADV (Fig. 3h, j–l). Only pSyn#64-IR neurites (thread-like structures) were observed in the mucosa (M) and muscularis mucosa (MM) (Fig. 3d, f). Mean percentages of the pathology were as follows: muscularis propria (MP), 41.6% (74/178); ADV, 33.1% (59/178); submucosa (SM), 14.6% (26/178); MM, 8.4% (15/178); M, 4.5% (8/178). The incidence increased with both BBAR and Braak LB-stage progression. MP and ADV exhibited the highest rates, reaching 89.5% (17/19) by BBAR stage 5, and 92.9% (26/28) and 78.6% (22/28) for MP and ADV, respectively, by Braak stage 6. In contrast, Lewy pathology was observed less in the M and MM (Fig. 4a, b; Table 4a, b).

**Table 4:** Incidence of Lewy pathology in the esophageal wall (a) BBAR LB stage, (b) Braak LB stage

	Number of cases (Mean age at death)	M *n* (%)	MM *n* (%)	SM *n* (%)	MP *n* (%)	ADV *n* (%)
(a) BBAR LB stage						
0.5	29 (81.2, SD 9.6)	0 (0)	0 (0)	0 (0)	0 (0)	1 (3.4)
1	83 (82.9, SD 10.1)	0 (0)	1 (1.2)	3 (3.6)	21 (25.3)	16 (19.3)
2	20 (83.8, SD 9.5)	1 (5.0)	2 (10.0)	4 (20.0)	13 (65.0)	9 (45.0)
3	8 (84.0, SD 6.8)	1 (12.5)	2 (25.0)	2 (25.0)	6 (75.0)	5 (62.5)
4	19 (85.2, SD 8.2)	1 (5.3)	4 (21.1)	7 (36.8)	17 (89.5)	11 (57.9)
5	19 (86.9, SD 7.6)	5 (26.3)	6 (31.6)	10 (52.6)	17 (89.5)	17 (89.5)
Total	178	8 (4.5)	15 (8.4)	26 (14.6)	74 (41.6)	59 (33.1)
(b) Braak LB stage						
0	12 (81.2, SD 12.6)	0 (0)	0 (0)	0 (0)	0 (0)	0 (0)
1	23 (84.5, SD 7.7)	0 (0)	0 (0)	0 (0)	0 (0)	0 (0)
2	14 (79.8, SD 10.9)	0 (0)	0 (0)	0 (0)	2 (14.3)	1 (7.1)
3	57 (82.6, SD 9.8)	0 (0)	1 (1.8)	3 (5.3)	16 (28.1)	14 (24.6)
4	17 (85.2, SD 10.2)	1 (5.9)	1 (5.9)	2 (11.8)	9 (52.9)	8 (47.1)
5	27 (83.4, SD 9.9)	5 (18.5)	5 (18.5)	10 (37.0)	21 (77.8)	14 (51.9)
6	28 (86.0, SD 6.1)	2 (7.1)	8 (28.6)	11 (39.3)	26 (92.9)	22 (78.6)
Total	178	8 (4.5)	15 (8.4)	26 (14.6)	74 (41.6)	59 (33.1)

*BBAR* the Brain Bank for Aging Research, *LB* Lewy body, *SD* standard deviation, *M* mucosa, *MM* muscularis mucosa, *SM* submucosa, *MP* muscularis propria, *ADV* adventitia

## Discussion

The present study demonstrates the following: (1) one-third of older people exhibited Lewy pathology in the CNS and/or PNS (178/518: 34%); and (2) the incidence of Lewy pathology in the esophagus was 43.8% in LBD (78/178): 92.1% in PDD (Parkinson disease with dementia)/DLB (35/38, BBAR LB stage 4–5), 75% in PD (Parkinson disease without dementia) (6/8, stage 3), 35% in preclinical/prodromal LBD (36/103, stage 1–2), 3.4% in the earliest stage of LBD (1/29, stage 0.5), and zero in controls (0/340, stage 0), which correlated with the progression of the BBAR LB stage.

### Clinicopathological study based on autopsy-confirmed cohorts of older individuals

Since esophageal LBs were first reported in PD [68], more than 40 articles investigating the incidence of Lewy pathology of the human ENS have been published (summarized in Supplementary Table 4, online resource). The significance of this study is that our target is a geriatric cohort, including PDD/DLB, PD without dementia, preclinical/prodromal PD, and controls. This differs most of the previous research, which only analyzed patients with PD. Further, this study evaluated multiple locations in the PNS based on autopsy data from numerous older cohorts and was quality-controlled by board-certified general pathologists and a specialist in gastrointestinal systems. Brain Banks in Japan are based on general autopsies and have a stronger impact on the study of Lewy pathology than do specimens of surgical pathology because autopsy can both confirm the neuropathological diagnosis and reveal its distribution throughout the whole body.

Although it is not a population-based cohort, the BBAR is more generalizable to the public than research cohorts are. The pathological causes of death for the 518 BBAR individuals had incidences as follows: respiratory disease, 27% (140 cases); malignant neoplasm, 27% (138); cardiovascular disease, 19% (100); cerebrovascular disease, 6% (33). The clinical causes of death for older people in Japan from 2010 to 2017 had incidence percentages as follows: malignant neoplasm, 27–28%; cardiovascular disease, 16–17%; respiratory disease, 9–13%; cerebrovascular disease, 8–11%; senile decay, 4–8% (Portal Site of Official Statistics of Japan, https://www.e-stat.go.jp/). The proportions are similar to those of the BBAR, except that the BBAR had a higher percentage of respiratory disease because it was a hospital cohort, while the overall statistics for Japanese include senile decay. Furthermore, in Japan, more than 75% of people die in the hospital rather than in their own houses. For example, in 2017, among the 1.3 million people who died, 1.0 million died in the hospital. Thus, the Japanese hospital-based cohort might reflect the Japanese population.

### The PNS in LBD

The way in which Lewy pathology propagates has been advanced by Braak and colleagues [11–13] as well as our research group [73, 76]. Two entries/pathways are hypothesized: (1) a brainstem-ascending pathway propagating from the ENS to the brainstem, limbic, and neocortex, and (2) an olfactory-amygdala pathway propagating from the olfactory epithelium and olfactory bulb to the amygdala [35]. Although Braak’s hypothesis proposed the ENS to be the entry zone of Lewy pathology [10], whether the gastrointestinal system is the origin of PD remains up for discussion [50]. Thus, we required further study.

In the current study, none of the cases exhibited Lewy pathology in the esophagus but not in the CNS. Our results support multiple foci of Lewy pathology in the PNS [4, 49] and reconfirmed its strong connection with the brain stem [10–13]. The incidence of Lewy pathology was higher in the brainstem (133/178: 74.7% in the dorsal motor nucleus of the vagus and locus coeruleus) and the olfactory bulb (146/174: 83.9%) than in the esophagus (43.8%). When the pathology existed in the brainstem, the positive rate for the esophagus was 58.6% (78/133). In the PNS, when the pathology was observed in the heart, adrenal gland, or skin, 15–37% lacked deposition in the esophagus. Therefore, we can say that the distribution of Lewy pathology throughout the PNS is varied. However, how sampling approaches to PNS tissues could influence on the positivity of Lewy pathology is recently discussed on in vivo skin biopsies [15, 32]. Previous studies described the rostral-caudal or proximal-distal gradient of the pathology [24, 25] and mismatch of positivity in a patient [39]. We did not perform serial sections of PNS tissues in this study. Thus, sampling bias might have some influence on the results.

Our results also show that Lewy pathology of the esophagus is a predictive factor for LBD. The incidence and severity of the esophageal pathology correlated with the BBAR LB stage: from 3.4% in the earliest stage (stage 0.5, 1/29) to 94.7% in the latest stage (stage 5; PDD/DLB, neocortical form, 18/19). We suspect that Lewy pathology of the esophagogastric junction (EGJ) could contribute to gastroesophageal reflux. Thus, our results partially explain the frequent complications of aspiration pneumonia in the late stage of LBD. We also show that the incidence of Lewy pathology in the autonomic nerves of the anterior wall of the heart decreased with progression from stage 4 (94.7%, 18/19) to 5 (84.2%, 16/19). We speculate that this could be related to the decrease in nerve fibers as reported previously [27, 62, 63]. However, the decrease might only reflect the small numbers of high-stage groups. The autonomic ganglions, including the segment innervating the heart, show 100% positivity after stage 2. In the esophagus, the myenteric plexus has been reported not to show loss or degeneration of ganglion cells in PD [2, 68, 90]. Further systemic analyses are required.

Lewy pathology of the esophagus was influenced by the type of LBD as described in the 4^th^ DLB Consensus Guidelines. It was high in diffuse neocortical LBD (44/49, 89.7%) and low in amygdala-predominant LBD (1/29, 3.4%). The amygdala variant is thought to be secondary to AD [67, 69], and we use the term “olfactory-amygdala” because this type usually involves olfaction. AD with LB exhibits rare α-synuclein deposition in the ENS [4, 31, 33]. However, our study showed a case with Lewy pathology in the esophagus and one AD case with amygdala-predominant LBD and α-synuclein deposition in sympathetic ganglia. A few neuritic structures were observed via α-synuclein immunohistochemistry, although the amount of staining was very small or faint compared with the diffuse DLB cases. Because AD does not involve the PNS, further research may be required to determine whether or not the olfactory-amygdala variant is secondary to AD.

## Limitation of this study

This study has some limitations. (1) The examination of the entire ENS was practically impossible. (2) Each type of PNS tissue, including the esophagus, was evaluated using single slides. However, questionable cases were reexamined by other anti-phosphorylated and non-phosphorylated α-synuclein antibodies. (3) Autonomic symptoms were retrospectively studied in medical charts and not prospectively.

## Conclusion

We found that one-third of older individuals exhibited Lewy pathology, 43.8% of whom in the esophagus. Further, among the different regions of the PNS positive for the pathology, the highest correlation with disease progression (BBAR LB stage) was observed in the esophagus. Therefore, this study provides a pathological basis for the development of digestive dysfunctions in LBD and suggests that esophageal Lewy pathology is a predictive factor of LBD.

## Electronic supplementary material

Below is the link to the electronic supplementary material.

Supplementary file1 (PDF 788 kb)
