# Supplementary material for: Lewy pathology of the esophagus correlates with the progression of Lewy body disease: a Japanese cohort study of autopsy cases
Source: Acta Neuropathol. 2020 Nov 5;141(1):25–37. doi: 10.1007/s00401-020-02233-8 (PMC7785549; doi:10.1007/s00401-020-02233-8)
Supplement: Supplementary file 1 — Supplementary file1 (PDF 788 kb) [file 401_2020_2233_MOESM1_ESM.pdf]

Lewy pathology of the esophagus correlates with the progression of Lewy body disease: a Japanese cohort study of autopsy cases

Zen-ichi Tanei[1,2], Yuko Saito[1], Shinji Ito[3], Tomoyasu Matsubara[1,4], Atsuko Motoda[1,4], Mikihiro Yamazaki[1,5], Yasuhiro Sakashita[1,6], Ito Kawakami[1,7], Masako Ikemura[8], Shinya Tanaka[2,9], Renpei Sengoku[1,5], Tomio Arai[10], Shigeo Murayama[1,11]

[1]Department of Neurology and Neuropathology (the Brain Bank for Aging Research), Tokyo Metropolitan Geriatric Hospital and Institute of Gerontology, Tokyo, Japan

[2]Department of Cancer Pathology, Faculty of Medicine, Hokkaido University, Sapporo, Japan

[3]Department of Pathology, Toranomon Hospital, Tokyo, Japan

[4]Department of Clinical Neuroscience & Therapeutics, Hiroshima University Graduate School of Biomedical and Health Sciences, Hiroshima, Japan

[5]Department of Neurology, The Jikei University School of Medicine, Tokyo, Japan

[6]Department of Neurology and Neurobiology of Aging, Kanazawa University Graduate School of Medical Sciences, Kanazawa, Japan

[7]Dementia Research Project, Tokyo Metropolitan Institute of Medical Science, Tokyo, Japan

[8]Department of Pathology, Graduate School of Medicine, The University of Tokyo, Tokyo, Japan.

[9]Institute for Chemical Reaction Design and Discovery (WPI-ICReDD), Hokkaido University, Sapporo, Japan

[10]Department of Pathology, Tokyo Metropolitan Geriatric Hospital and Institute of Gerontology, Tokyo, Japan

[11]Brain Bank for Neurodevelopmental, Neurological and Psychiatric Disorders, United Graduate School of Child Development, Osaka University, Osaka, Japan

Correspondence:

Shigeo Murayama, M.D., Ph.D.

Department of Neurology and Neuropathology (the Brain Bank for Aging Research)

Tokyo Metropolitan Geriatric Hospital and Institute of Gerontology

35-2 Sakae-cho, Itabashi-ku, Tokyo 173-0015, Japan.

Tel: +81-3-3964-1141; Fax: +81-3-3579-4776; E-mail: smurayam@bbarjp.net

Supplementary materials

Supplementary Figure 1 Immunohistochemistry using four different anti-α-synuclein antibodies. (a) LB in Auerbach's plexus of the esophagus. (b–e) Immunoreactivity of LB and neuronal processes observed in four anti-α-synuclein antibodies. H&E (a), pSyn#64 (b), MJF-R13 (c), LB509 (d), and PSer129 (e). Scale bar = 100 μm.

Supplementary Figure 2 Cause of death for the 518 BBAR cases at autopsy. Three-fourths of the individuals died of respiratory diseases, malignant neoplasm, or cardiovascular disease. Dotted areas in the inner circle show comorbidity with neurodegenerative diseases, such as AD, PD, PDD, DLB, argyrophilic grain disease, neurofibrillary tangle predominant dementia, frontotemporal lobar degeneration, progressive supranuclear palsy, or others.

Supplementary Figure 3 List of the 178 BBAR cases who showed Lewy pathology in their nervous systems. Semi-quantitative analyses of the α-synuclein deposition are colored in blue gradation (score 0–4 according to DLB Consensus guidelines). *AA*, amyloid angiopathy; *AAD*, acute aortic dissection; *AAneu*, aortic aneurysm; *AD*, Alzheimer's disease; *Adr*, adrenal gland; *AF*, autonomic failure; *Af* , atrial fibrillation; *AGD*, argyrophilic grain disease; *ALS*, amyotrophic lateral sclerosis; *AMI*, acute myocardial infarction; *AML*, acute myeloid leukemia; *Amy*, amygdala; *AON*, anterior olfactory nucleus; *AS*, aortic valve stenosis; *BBAR LB*, Lewy body stage of the Brain Bank for Aging Research; *BT*, brain tumor; *Braak LB*, Braak Lewy body stage; *BH*, brain hemorrhage; *BI*, brain infarction; *BW*, brain weight (g); CERAD, 0 = none, A = sparse, B = moded, C = frequent; *ca*, cancer; *CBS*, corticobasal syndrome; *CHF*, congestive heart failure; *Cing*, anterior cingulate gyrus; *CML*, chronic myeloid leukemia; *COPD*, chronic obstructive pulmonary disease; *CRF*, chronic renal failure; *CVD*, cerebrovascular disease; *DLB*, dementia with Lewy bodies; *DM*, diabetes mellitus; *DMNV*, dorsal motor nucleus of the vagus; *DLB 4th*, fourth consensus report of the DLB consortium; *DLBN*, dementia with Lewy bodies, neocortical form; *DLBT*, dementia with Lewy bodies, transitional form; *DNTC*, diffuse neurofibrillary tangles with calcification; *Eso*, esophagus; *Esophagus M*, mucosa of the esophagus; *Esophagus MM*, muscularis mucosa; *Esophagus SM*, submucosa; *Esophagus MP*, muscularis propria; *Esophagus ADV*, adventitia; *F*, frontal lobe; *FTLD*, frontotemporal lobar degeneration; *GI bleed*, gastrointestinal bleeding; *Grain*, Saito grain stage; *HCM*, hypertrophic cardiomyopathy; *He*, heart; *HY*, Hoehn and Yahr scale; *IE*, infective endocarditis; *IHC*, immunohistochemistry, ✔ = performed; *IHD*, ischemic heart disease; *Int Perf*, intestinal perforation; *IPneu*, interstitial pneumonia; *IVL*, intravascular lymphoma; *LBD*, Lewy body disease; *LC*, locus coeruleus; *M*, medullar oblongata; *MDD*, major depressive disorder; *MDS*, myelodysplastic syndrome; *ML*, malignant lymphoma; *MM*, multiple myeloma; *MPA*, microscopic polyangiitis; *NA*, not available; *nbM*, nucleus basalis of Meynert; *NFT*, Braak neurofibrillary tangle stage; *NFTD*, neurofibrillary tangle predominant dementia; *NPD*, neuropathological diagnosis; *OB*, olfactory bulb; *P*, parietal lobe; *PA*,

Parkinsonism. 1 = presence, 0 = absence; *PAF*, pure autonomic failure; *Peri*, the periphery of the OB; *PD*, Parkinson's disease; *PDD*, Parkinson's disease with dementia; *Pneu*, pneumonia; *PSP*, progressive supranuclear palsy; *RA*, rheumatoid arthritis; *RF*, respiratory failure; *S*, lumbar spinal cord; *SAH*, subarachnoid hemorrhage; *SCA*, spinocerebellar ataxia; *SDH*, subdural hemorrhage; *SN*, substantia nigra; *SP*, Braak senile plaque stage; *SY*, sympathetic ganglia; *T*, temporal lobe; *Thal*, Thal senile plaque phase; *TrE*, transentorhinal cortex; *UP*, unknown primary.

Supplementary Figure 1 Immunohistochemistry using four different anti-α-synuclein antibodies

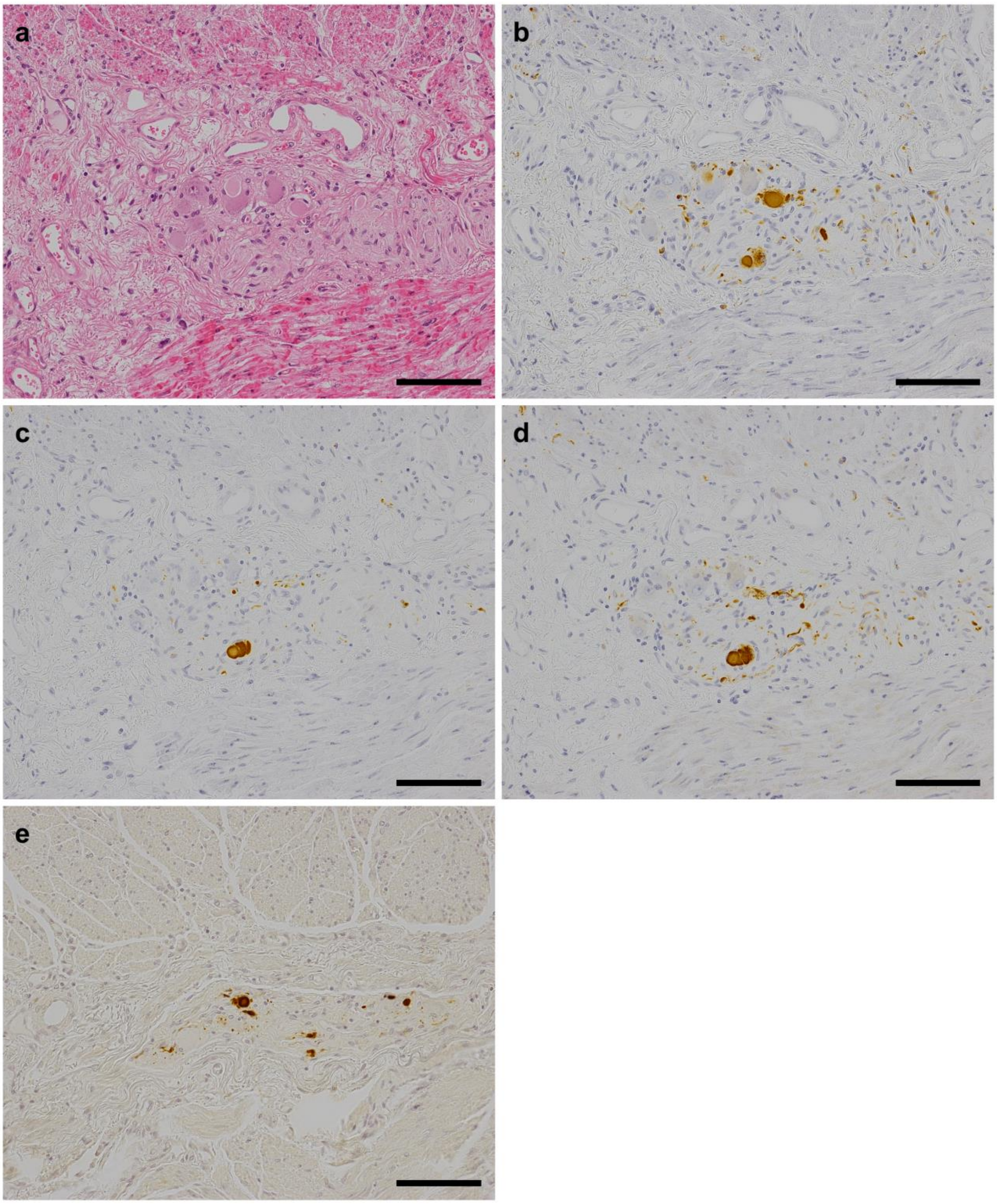

Supplementary Figure 2 Cause of death for the 518 BBAR cases at autopsy

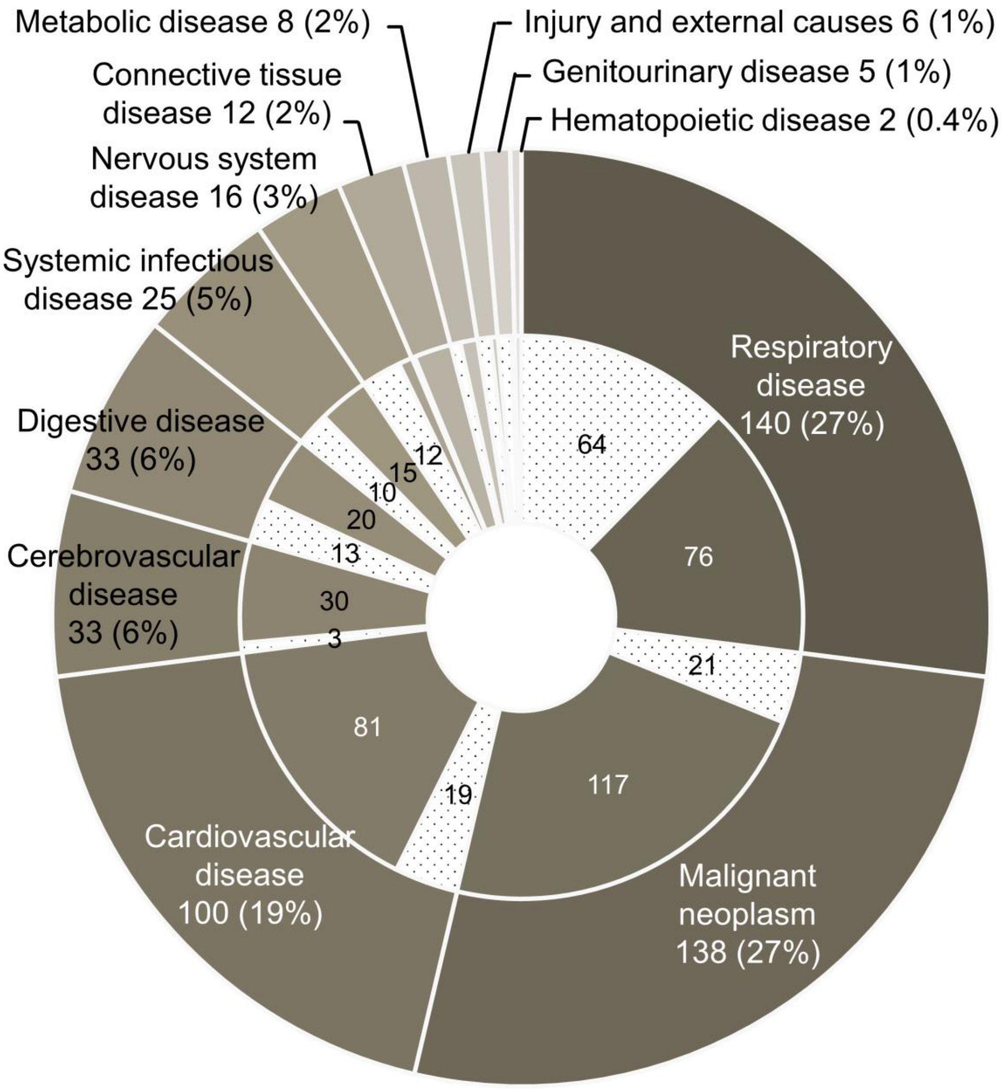

Supplementary Figure 3 List of the 178 BBAR cases who showed Lewy pathology in their nervous systems

BBAR LB	DLB 4th	Braak LB	Age	Sex	AF	PA	Clinical diagnosis	NPD	BW	ApoE	NFT	SP	CERAD	Thal	Grain	pTDP43 T	pTDP43 M	pTDP43 S	DMNV	LC	SN	nbM	Amy	TrE	Cing	T	F	P	OB Peri	OB AON	Skin	Adr	He	Eso	SY	Esophagus M	Esophagus MM	Esophagus SM	Esophagus MP	Esophagus AD	IHC for esophagus pSyn#64	IHC for esophagus PSer129	IHC for esophagus MJF-R13	IHC for esophagus LB509
0.5OB	Olfactory bulb only	1	94	M	0	0	Pneu	CVD	1267	33	3	2	B	4	2	1	1	1	0	0	0	0	0	0	0	0	0	0	1	0	0	0	0	0	0	0	0	0	0	0	✔			
0.5OB	Olfactory bulb only	1	84	F	0	0	Duodenal ca, DM	CVD	1065	34	3	2	C	4	2	1	1	1	0	0	0	0	0	0	0	0	0	0	1	0	0	0	0	0	0	0	0	0	0	0	✔			
0.5OB	Olfactory bulb only	1	88	M	1	1	AD, DLB, Pneu	AD	1220	33	5	3	C	4	0.5	0	0	0	0	0	0	0	0	0	0	0	0	0	1	1	0	0	0	0	0	0	0	0	0	0	✔			
0.5SY	PNS only	0	96	F	0	0	Sepsis	NFTD & CVD	1032	33	3	1	A	2	2	0	0	1	0	0	0	0	0	0	0	0	0	0	0	0	0	0	0	0	1	0	0	0	0	0	✔	✔		
0.5SY	PNS only	0	85	F	0	0	CHF	CVD	1183	33	2	1	A	1	1	0	0	0	0	0	0	0	0	0	0	0	0	0	0	0	0	0	0	0	1	0	0	0	0	0	✔			
0.5SY	PNS only	0	74	M	0	0	Ca of UP	CVD	1318	33	1	1	A	1	0.5	1	2	0	0	0	0	0	0	0	0	0	0	0	0	0	0	0	0	0	1	0	0	0	0	0	✔	✔		✔
0.5A	Amygdala-predominant	0	83	M	0	0	Pneu, SAH	AD	1321	33	4	3	C	5	1	0	0	0	0	0	0	0	1	0	0	0	0	0	0	0	0	0	0	0	0	0	0	0	0	0	✔			
0.5A	Amygdala-predominant	1	85	F	0	0	Lung ca	Metastatic tumor	1030	34	3	1	A	1	2	3	0	0	0	0	0	0	1	0	0	0	0	0	1	0	0	0	0	0	0	0	0	0	0	0	✔			
0.5A	Amygdala-predominant	1	81	F	0	0	Lung ca	Metastatic tumor & CVD	1271	34	3	3	C	5	1	2	1	1	0	0	0	0	1	0	0	0	0	0	0	1	0	0	0	0	0	0	0	0	0	0	✔			
0.5A	Amygdala-predominant	0	86	M	0	0	Pneu	AD & AA	1219	33	5	3	C	5	1	0	0	0	0	0	0	0	1	1	0	0	0	0	0	0	0	0	0	0	0	0	0	0	0	0	✔			
0.5A	Amygdala-predominant	3	92	F	0	0	COPD	AD change	1072	33	4	2	C	4	1	1	0	1	0	0	0	1	1	0	1	0	0	0	1	0	0	0	0	0	0	0	0	0	0	0	✔			
0.5A	Amygdala-predominant	1	90	F	0	0	IPneu	SDH	1206	23	2	1	A	1	0.5	1	0	0	0	0	0	0	1	0	0	0	0	0	1	0	0	0	0	0	1	0	0	0	0	0	✔			
0.5A	Amygdala-predominant	0	89	F	1	1	AD, BI	AD & CVD	1169	34	5	3	C	5	1	0	0	0	0	0	0	0	1	0	0	0	0	0	0	0	0	0	0	0	0	0	0	0	0	0	✔			
0.5A	Amygdala-predominant	1	72	M	0	0	Urinary bladder ca	Earliest LBD	1236	34	2	1	A	2	1	1	0	0	0	0	0	0	1	0	0	0	0	0	1	1	0	0	0	0	0	0	0	0	0	0	✔			
0.5A	Amygdala-predominant	1	76	M	0	0	Stomach ca	CVD	1165	33	3	3	B	4	0.5	0	0	0	0	0	0	0	1	0	0	0	0	0	1	1	0	0	1	0	1	0	0	0	0	0	✔			
0.5B	Brainstem-predominant	3	83	F	0	0	RA	Cervical spondylosis	1046	33	3	0	0	0	1	0	0	0	1	1	1	1	0	0	0	0	0	0	0	0	0	0	2	1	1	0	0	0	0	1	✔			
0.5B	Brainstem-predominant	1	94	F	0	0	Stomach ca	NFT dominant change	1143	33	3	1	A	3	2	2	1	0	1	0	0	0	0	0	0	0	0	0	0	0	0	0	0	0	0	0	0	0	0	0	✔			
0.5B	Brainstem-predominant	2	72	M	0	0	OMI, DM	Earliest LBD	1070	33	1	1	0	1	0	1	0	0	0	1	0	0	0	0	0	0	0	0	0	0	0	0	0	0	1	0	0	0	0	0	✔			
0.5B	Brainstem-predominant	2	71	M	1	1	Pneu	Earliest LBD	1360	33	1	1	0	4	0.5	0	0	0	1	1	0	0	0	0	0	0	0	0	0	0	0	0	0	0	0	0	0	0	0	0	✔			
0.5B	Brainstem-predominant	2	68	M	0	0	AML	Meningoencephalitis	1244	33	2	1	0	1	0.5	0	0	0	1	1	0	0	0	0	0	0	0	0	0	0	0	0	0	0	0	0	0	0	0	0	✔			
0.5B	Brainstem-predominant	3	72	M	1	0	ALS	ALS	1403	33	1	1	0	1	2	3	3	3	0	1	1	0	0	0	0	0	0	0	0	0	0	0	0	0	0	0	0	0	0	0	✔			
0.5B	Brainstem-predominant	3	63	M	0	0	Prostate ca	Metastatic tumor	1358	34	0	1	0	2	0	0	0	0	1	1	0	1	0	0	0	0	0	0	0	0	0	0	0	0	0	0	0	0	0	0	✔			
0.5L	Brainstem-predominant	1	87	F	0	0	BI	CVD	1090	33	3	1	A	1	2	0	0	1	0	0	1	0	0	0	0	0	0	0	1	1	0	0	0	0	0	0	0	0	0	0	✔			
0.5L	Brainstem-predominant	2	89	M	1	0	CML	Cervical spondylosis	1057	33	2	1	A	1	2	2	1	0	0	1	0	0	1	0	0	0	0	0	1	0	0	0	0	0	0	0	0	0	0	0	✔			
0.5L	Amygdala-predominant	3	83	M	0	0	Pneu	AD & CVD	1205	34	5	3	C	5	2	2	0	0	0	0	1	0	1	1	0	0	0	0	1	0	0	0	0	0	0	0	0	0	0	0	✔			
0.5L	Brainstem-predominant	2	83	F	0	0	BH	CVD	1187	34	2	3	C	5	0.5	0	0	0	1	1	0	0	1	0	1	0	0	0	NA	NA	0	0	0	0	0	0	0	0	0	0	✔			
0.5L	Brainstem-predominant	3	61	M	0	0	Encephalitis	Meningoencephalitis	1442	33	1	0	0	0	0	0	1	0	1	0	1	0	1	0	0	0	0	0	1	0	0	0	0	0	0	0	0	0	0	0	✔			
0.5L	Brainstem-predominant	3	67	F	0	0	AAD	CVD	1338	33	2	1	0	1	0	1	0	1	1	1	1	0	1	0	0	0	0	0	0	0	1	0	1	0	1	0	0	0	0	0	✔			
0.5L	Brainstem-predominant	3	87	M	0	0	BI	CVD & AA	1285	33	2	3	B	5	0.5	1	1	1	1	1	0	1	1	0	0	0	0	0	1	1	0	2	0	0	3	0	0	0	0	0	✔			
1Heart	PNS only	0	83	F	0	0	Pneu, OMI	AGD	1123	33	3	1	A	1	3	0	0	0	0	0	0	0	0	0	0	0	0	0	0	0	0	0	2	0	1	0	0	0	0	0	✔			
1Heart	PNS only	0	71	M	0	0	ML	Preclinical LBD	1507	33	1	1	A	1	1	1	0	0	0	0	0	0	0	0	0	0	0	0	0	0	0	0	2	0	0	0	0	0	0	0	✔			
1SY	Brainstem-predominant	1	69	F	0	0	IHD, DM	NFTD & CVD	1264	33	4	0	0	0	1	0	0	0	1	0	0	0	0	0	0	0	0	0	0	0	0	2	1	0	3	0	0	0	0	0	✔	✔		
1SY	Brainstem-predominant	2	70	M	0	0	Pneumoconiosis	Preclinical LBD	1277	23	1	1	A	1	0	1	0	0	1	1	0	0	0	0	0	0	0	0	0	0	1	1	1	1	3	0	0	0	1	0	✔	✔		
1SY	PNS only	0	89	M	0	0	BT	Contusion	1122	33	2	2	B	2	0.5	1	1	0	0	0	0	0	0	0	0	0	0	0	0	0	0	0	2	0	2	0	0	0	0	0	✔	✔		
1SY	PNS only	0	93	M	1	0	Pneu	SDH	1122	23	3	3	C	3	1	1	0	0	0	0	0	0	0	0	0	0	0	0	0	0	0	0	0	0	2	0	0	0	0	0	✔			
1SY	PNS only	0	49	M	0	0	OMI, DM, CRF	CVD	1465	33	1	0	0	0	0	0	0	0	0	0	0	0	0	0	0	0	0	0	0	0	0	0	0	0	3	0	0	0	0	0	✔			
1SY	PNS only	0	77	F	0	0	Ileus	Preclinical LBD	1252	33	2	0	0	0	2	1	0	0	0	0	0	0	0	0	0	0	0	0	0	0	0	0	1	0	3	0	0	0	0	0	✔			
1A	Amygdala-predominant	1	96	F	0	0	AD, Pneu	FTLD-TDP	1138	23	2	0	0	0	2	3	2	0	0	0	0	0	1	0	0	0	0	0	2	2	0	0	0	0	0	0	0	0	0	0	✔			
1A	Amygdala-predominant	1	92	M	0	0	CRF	AD	1235	34	3	3	C	5	2	0	0	0	0	0	0	0	2	0	0	0	0	0	2	1	0	0	0	0	0	0	0	0	0	0	✔			
1A	Amygdala-predominant	3	78	M	0	0	IPneu	AGD	1400	34	2	1	A	1	3	0	0	0	1	2	1	1	3	0	0	0	0	0	2	2	0	0	2	0	1	0	0	0	0	0	✔	✔		
1A	Amygdala-predominant	3	72	M	0	0	MDS	Brain abscess	1295	33	1	1	A	1	0.5	0	0	0	0	0	1	2	1	0	0	0	0	0	2	1	0	0	0	0	0	0	0	0	0	0	✔			
1A	Amygdala-predominant	3	94	M	0	0	Pneu	NFTD	1101	33	4	1	A	3	1	0	0	0	0	0	0	1	1	1	0	0	0	0	2	1	0	0	0	0	0	0	0	0	0	0	✔	✔		
1A	Amygdala-predominant	3	86	M	0	0	Pneu, BH	CVD	1345	33	2	1	A	3	3	0	1	1	1	1	1	1	2	0	0	1	0	0	1	2	0	0	0	0	1	0	0	0	0	0	✔	✔		
1A	Amygdala-predominant	1	85	M	0	0	MM	NFT dominant change	1351	33	3	1	A	3	2	1	1	1	0	0	0	0	2	0	0	0	0	0	2	1	0	0	0	0	0	0	0	0	0	0	✔			
1A	Amygdala-predominant	1	81	F	0	1	PD (HY5), Pneu	AD & PML	1184	33	6	3	C	5	1	2	1	1	0	0	0	0	2	0	0	0	0	0	1	2	0	0	0	0	0	0	0	0	0	0	✔			
1A	Amygdala-predominant	1	77	M	0	0	IPneu	CVD	1158	34	2	1	A	3	0.5	1	1	1	0	0	0	0	1	0	0	0	0	0	2	1	0	0	0	0	0	0	0	0	0	0	✔			
1A	Amygdala-predominant	1	78	M	0	1	AD, Pneu	AD	1116	44	6	3	C	5	1	2	0	0	0	0	0	0	3	0	1	1	0	0	2	4	0	0	0	0	0	0	0	0	0	0	✔			
1A	Amygdala-predominant	1	91	M	0	0	AD, Pneu	AD & AA & CVD	1341	33	6	3	C	5	2	3	0	0	0	0	0	0	2	1	1	0	0	0	3	1	0	0	0	0	0	0	0	0	0	0	✔			
1A	Amygdala-predominant	3	99	F	0	0	AD, AMI	AD & CVD	1000	34	6	3	C	5	1	3	1	1	0	0	2	1	3	2	1	1	0	0	2	1	0	0	0	0	0	0	0	0	0	0	✔			
1A	Amygdala-predominant	3	90	F	0	0	SAH, DM	SAH	1161	33	3	1	A	1	0	1	0	0	0	0	1	1	2	0	0	0	0	0	2	0	0	0	0	0	0	0	0	0	0	0	✔			
1A	Amygdala-predominant	3	90	F	0	0	AD, Pneu	AD & AGD	916	33	5	3	C	5	3	3	2	1	0	0	0	1	3	1	1	0	0	0	1	1	0	0	0	0	0	0	0	0	0	0	✔			
1A	Amygdala-predominant	3	87	F	0	0	Pneu	CVD	1009	34	3	1	B	1	2	1	0	0	0	0	0	1	2	0	0	0	0	0	1	1	0	0	0	0	0	0	0	0	0	0	✔			
1A	Amygdala-predominant	1	91	F	0	0	AD, Liver cirrhosis	AD	936	33	6	3	C	5	1	3	1	0	0	0	0	0	1	0	0	0	0	0	1	2	0	0	0	0	0	0	0	0	0	0	✔			
1A	Amygdala-predominant	2	88	F	0	0	Shock	AGD	1209	33	3	3	B	5	2	3	0	0	1	1	0	0	2	0	0	0	0	0	3	2	0	2	1	1	2	0	0	0	0	1	✔			
1A	Amygdala-predominant	1	89	F	0	0	AD, CHF	AD	1088	33	5	3	C	5	0.5	2	0	1	0	0	0	0	1	0	0	0	0	0	1	2	0	0	0	0	1	0	0	0	0	0	✔			
1A	Amygdala-predominant	1	87	F	0	0	AD, BT	Glioblastoma & AD change	1232	33	3	3	C	5	1	2	1	1	0	0	NA	NA	1	1	0	0	0	0	2	0	0	0	0	0	0	0	0	0	0	0	✔			
1B	Brainstem-predominant	3	89	F	0	0	AMI	CVD	1354	33	3	3	C	5	0.5	0	0	0	3	3	1	1	1	0	0	0	0	0	2	2	0	0	2	1	2	0	0	0	1	1	✔			
1B	Brainstem-predominant	2	97	F	0	0	Stomach ca, BI	CVD	1254	33	2	1	A	3	0.5	0	0	1	0	2	0	0	0	0	0	0	0	0	1	0	0	0	0	0	0	0	0	0	0	0	✔			
1B	Brainstem-predominant	3	78	M	0	0	Pneu, OMI	Preclinical LBD	1274	33	1	1	A	1	0.5	0	0	0	3	2	1	1	1	0	1	0	0	0	2	1	0	2	1	2	4	0	0	0	2	0	✔			
1B	Brainstem-predominant	3	79	M	0	0	Lung ca	Metastatic tumor	1195	23	1	1	A	1	1	0	0	0	1	2	1	1	1	0	0	0	0	0	2	1	0	0	2	0	2	0	0	0	0	0	✔			
1B	Brainstem-predominant	2	90	F	0	0	Pneu, BI	CVD	1118	23	2	1	A	1	1	1	0	0	0	2	0	1	1	0	0	0	0	0	2	1	0	0	0	0	0	0	0	0	0	0	✔			
1B	Brainstem-predominant	3	81	F	0	0	SCA3, Pneu	Machado-Joseph disease	1190	33	2	1	A	1	0.5	0	0	0	3	2	1	1	2	0	0	0	0	0	2	2	0	2	0	1	4	0	0	0	1	1	✔			
1B	Brainstem-predominant	3	68	M	0	0	Lung ca	Metastatic tumor	1114	33	2	0	0	0	0	0	0	0	1	3	1	1	0	0	0	0	0	0	1	2	1	2	2	1	3	0	0	0	0	1	✔	✔		
1B	Brainstem-predominant	3	81	M	0	0	Pneu	CVD	1330	33	2	0	0	0	0.5	0	0	0	2	2	1	1	1	0	1	0	0	0	1	1	0	0	0	0	0	0	0	0	0	0	✔			
1B	Brainstem-predominant	2	57	M	0	0	AML, Pneu	Preclinical LBD	1253	33	0	1	A	1	0.5	0	0	0	2	1	0	0	0	0	0	0	0	0	0	0	0	0	1	0	0	0	0	0	0	0	✔			
1B	Brainstem-predominant	3	82	M	0	0	BI, Pneu	CVD	1328	33	1	1	A	1	0.5	1	0	0	2	2	1	0	1	0	0	0	0	0	2	0	0	2	2	0	3	0	0	0	0	0	✔			
1B	Brainstem-predominant	2	87	F	0	0	AD, HCM	CBD	1051	33	3	3	C	3	3	3	0	0	2	2	0	0	1	0	0	0	0	0	0	1	0	0	0	1	3	0	0	0	1	0	✔			
1B	Brainstem-predominant	3	98	M	0	0	AS	NFT dominant change	1185	33	3	1	A	3	0.5	3	0	0	1	1	1	2	1	0	0	0	0	0	1	1	0	0	0	1	1	0	0	0	1	0	✔			
1B	Brainstem-predominant	1	71	M	0	0	DM	SAH	1386	33	1	1	A	1	0.5	2	0	1	2	0	0	0	0	0	0	0	0	0	0	0	0	1	3	0	3	0	0	0	0	0	✔	✔		
1B	Brainstem-predominant	3	96	M	0	0	Pneu	Preclinical LBD	1160	33	2	1	0	1	0	1	0	0	2	2	1	1	1	1	0	0	0	0	2	2	0	0	1	2	3	0	0	1	2	1	✔			
1B	Brainstem-predominant	2	85	M	0	0	IPneu	CVD	1185	33	3	3	B	3	2	2	0	0	2	1	0	0	0	0	0	0	0	0	0	0	0	0	0	0	0	0	0	0	0	0	✔			
1B	Brainstem-predominant	3	72	M	0	0	BT	Glioblastoma	1273	33	2	1	0	1	2	0	0	1	3	0	1	0	0	0	0	0	0	0	1	0	0	2	1	0	3	0	0	0	0	0	✔			✔
1B	Brainstem-predominant	2	83	M	0	0	BT, DM	Primary CNS lymphoma	1264	33	1	0	0	0	1	0	0	0	2	1	0	1	0	0	0	0	0	0	0	0	0	0	0	0	1	0	0	0	0	0	✔			
1B	Brainstem-predominant	3	80	F	0	1	PSP	PSP	1026	34	2	1	A	2	0.5	1	0	0	1	2	2	1	1	1	0	0	0	0	2	1	0	2	0	1	2	0	0	0	1	1	✔			
1B	Brainstem-predominant	3	74	M	0	0	MDS	Preclinical LBD	1188	33	3	1	0	2	1	0	0	0	1	2	1	1	1	1	1	0	0	0	0	0	0	0	0	0	0	0	0	0	0	0	✔			
1B	Brainstem-predominant	3	87	M	0	0	IPneu	HSV-1 meningoencephalitis	1224	33	3	1	B	2	2	1	0	0	1	1	1	1	1	0	0	0	0	0	4	3	0	0	0	0	0	0	0	0	0	0	✔			
1B	Brainstem-predominant	2	77	F	0	0	Af, BI	CVD	1012	NA	2	3	B	4	0.5	1	0	0	1	2	0	0	1	0	1	0	0	0	NA	NA	0	0	0	0	0	0	0	0	0	0	✔			
1B	Brainstem-predominant	1	86	M	0	0	Pancreatic ca	SDH	1319	34	2	3	B	4	0.5	0	0	0	2	0	0	0	0	0	0	0	0	0	0	0	0	0	1	0	3	0	0	0	0	0	✔			
1B	Brainstem-predominant	3	72	M	0	0	Lung ca, DM	Metastatic tumor	1481	33	1	1	0	2	0	0	0	2	2	2	1	1	1	1	0	1	0	0	2	1	0	2	1	2	3	0	0	0	2	1	✔			
1B	Brainstem-predominant	3	94	F	0	0	MPA	CVD	1132	33	2	1	0	1	1	1	0	1	1	2	2	1	2	1	1	0	0	0	2	1	0	0	0	0	1	0	0	0	0	0	✔			
1B	Brainstem-predominant	3	102	F	0	0	Pneu	Epileptic encephalopathy	1041	33	3	1	A	2	0	0	0	0	1	2	1	1	1	0	0	0	0	0	1	0	0	0	1	0	2	0	0	0	0	0	✔			
1B	Brainstem-predominant	3	79	F	0	0	Stomach ca	NFT dominant change	1174	33	2	1	0	0	0.5	0	0	1	2	1	1	1	1	0	0	0	0	0	2	1	0	0	0	0	1	0	0	0	0	0	✔			
1L	Limbic	4	75	M	0	0	OMI, DM	Preclinical LBD	1251	33	2	1	A	1	0.5	0	0	1	3	3	1	3	3	2	2	1	1	0	2	1	0	0	1	2	3	0	0	0	2	1	✔			
1L	Limbic	3	82	M	0	0	Pneu	PSP change	1215	33	1	1	A	1	1	0	0	0	1	1	1	2	3	2	1	0	0	0	2	3	0	0	0	0	0	0	0	0	0	0	✔			
1L	Diffuse neocortical	5	100	F	0	0	IE	Meningitis	1100	33	4	2	B	5	2	3	2	1	3	3	2	3	4	3	3	2	2	2	2	4	0	0	3	1	3	0	0	0	1	0	✔			
1L	Limbic	4	87	M	0	0	Liver cirrhosis	CVD	1314	33	1	1	A	1	0.5	1	0	0	3	2	2	3	4	3	2	0	1	0	2	2	0	0	2	0	2	0	0	0	0	0	✔			
1L	Limbic	3	83	M	0	0	COPD	Preclinical LBD	1053	33	3	1	C	4	1	0	1	0	2	2	2	2	4	2	1	0	1	1	2	4	0	0	1	0	0	0	0	0	0	0	✔			
1L	Limbic	3	84	M	0	0	Liver failure	SDH	1184	34	2	3	C	5	3	0	0	0	3	3	2	3	4	2	2	1	1	1	3	4	0	0	2	0	3	0	0	0	0	0	✔			
1L	Limbic	3	86	F	0	0	BI	CVD	1225	33	NA (<=2)	3	B	3	2	0	0	0	2	2	2	1	1	1	2	1	1	1	1	3	0	1	1	0	3	0	0	0	0	0	✔			
1L	Limbic	3	89	M	0	0	Pneu	CVD	1254	24	2	2	C	4	0.5	0	0	0	3	3	2	2	4	2	2	1	1	1	2	4	0	2	1	1	3	0	0	0	1	1	✔			
1L	Limbic	3	78	M	0	0	IHD	CVD	1306	33	1	0	0	0	0.5	0	0	0	3	2	2	2	2	1	2	0	0	0	2	2	0	0	1	1	2	0	1	1	1	0	✔			
1L	Limbic	4	87	M	0	0	AML	Preclinical LBD	1438	33	3	1	A	1	1	2	0	0	3	3	1	3	3	2	2	1	0	0	1	2	0	0	1	0	2	0	0	0	0	0	✔			
1L	Limbic	3	94	F	0	0	Int Perf	Preclinical LBD	1068	33	2	1	A	1	1	0	1	0	2	2	1	3	3	1	2	2	1	1	2	2	0	1	1	2	3	0	0	0	2	1	✔			
1L	Limbic	3	97	M	0	1	AGD, Pneu	NFTD	1137	33	4	1	B	3	2	1	0	0	2	1	2	2	3	1	2	1	1	0	1	2	0	0	1	0	1	0	0	0	0	0	✔	✔		✔
1L	Limbic	6	81	F	0	0	Sepsis	AD	1188	33	5	3	C	4	0	1	0	0	1	2	2	2	3	3	1	2	2	1	2	4	1	0	1	2	3	0	0	0	2	1	✔			
1L	Limbic	3	76	M	0	0	AAneu, CRF	SDH	1479	33	2	0	0	0	2	1	0	0	2	2	2	2	3	2	1	0	0	0	2	2	0	3	0	1	3	0	0	0	1	0	✔			✔
1L	Limbic	4	61	M	1	0	MM	Preclinical LBD	1434	34	0	1	A	1	0	0	0	0	2	2	1	3	3	1	2	1	0	0	1	1	0	0	1	0	1	0	0	0	0	0	✔			✔
1L	Limbic	3	65	M	0	0	Stomach ca	Preclinical LBD	1402	33	1	1	0	1	0.5	0	0	0	1	1	1	2	3	1	1	1	1	1	1	0	0	0	0	0	0	0	0	0	0	0	✔			✔
1L	Limbic	3	82	M	0	0	AD, Pneu	AD	1121	33	5	3	B	5	1	3	0	0	1	1	1	1	3	1	1	1	1	1	1	0	1	0	0	0	0	0	0	0	0	0	✔			✔
1L	Limbic	3	95	F	0	0	AD, Pneu	Preclinical LBD	1042	33	2	2	B	2	1	0	0	0	1	1	1	1	2	1	1	1	1	1	1	1	0	0	0	0	0	0	0	0	0	0	✔			✔
1L	Limbic	3	86	F	0	0	BI, IHD, DM	CVD	1126	33	3	3	B	5	1	0	0	0	1	1	1	1	2	1	1	1	1	1	NA	NA	0	0	0	0	0	0	0	0	0	0	✔			
1L	Limbic	3	85	F	0	0	Pneu	Preclinical LBD	1349	33	2	1	0	1	1	1	0	0	1	2	2	2	2	2	2	1	1	1	1	2	0	1	1	0	1	0	0	0	0	0	✔			✔
1L	Limbic	3	84	M	0	0	AD, Stomach ca	AD	1210	34	5	3	C	4	1	1	0	0	2	2	2	2	3	1	1	1	1	1	3	3	0	0	0	1	3	0	0	0	1	1	✔			
1L	Limbic	3	87	F	0	0	AD, Pneu	AD	1115	34	6	3	C	5	1	1	1	0	1	1	1	1	3	1	1	1	0	0	3	3	0	0	0	0	0	0	0	0	0	0	✔			
1L	Limbic	3	75	M	0	0	Kidney ca	Metastatic tumor	1340	33	2	1	0	1	0.5	1	0	1	3	3	2	1	3	1	1	1	1	1	2	2	0	1	2	1	2	0	0	0	1	1	✔			
1L	Limbic	3	97	F	0	0	IPneu	CVD	1229	33	2	1	A	2	1	1	0	1	2	3	2	2	4	2	1	1	1	1	1	0	1	0	1	0	3	0	0	0	0	0	✔			
1L	Limbic	3	61	F	0	0	AD, AML	Tauopathy	1400	33	3	0	0	0	0.5	0	1	0	1	1	1	2	3	1	1	0	0	0	1	0	0	0	0	0	0	0	0	0	0	0	✔			
1L	Limbic	3	82	M	0	0	Lung ca	Preclinical LBD	1236	34	3	3	B	4	0.5	0	0	0	2	2	1	2	2	2	2	1	1	1	2	2	1	2	1	2	3	0	0	2	2	2	✔			
1L	Limbic	3	84	M	0	0	ALS	ALS	1363	33	3	1	0	1	0.5	0	3	3	2	2	2	2	2	1	1	1	0	1	2	2	0	0	1	0	2	0	0	0	0	0	✔			
1L	Limbic	3	86	M	1	0	Tuberculosis	Preclinical LBD	1213	23	1	1	0	1	0.5	1	1	1	3	3	2	2	2	1	2	0	0	0	2	2	1	1	0	1	2	0	0	0	1	1	✔			
1L	Limbic	3	80	M	0	0	AD, Pneu	AD & AGD	1340	33	6	3	C	5	3	1	0	0	1	1	1	1	2	1	0	1	0	0	2	2	1	0	1	0	3	0	0	0	0	0	✔			
1L	Limbic	3	74	M	1	0	ML	Contusion	1227	33	1	1	0	1	0.5	1	0	0	3	3	2	2	2	2	2	1	0	1	1	1	0	1	1	1	3	0	0	0	1	1	✔			
2	Limbic	4	68	F	0	0	IHD, DM	Prodromal LBD	1116	33	2	1	A	1	1	0	0	0	3	3	2	2	2	2	2	0	0	0	3	2	0	1	1	2	4	0	0	0	2	0	✔			
2	Limbic	5	86	M	0	0	Pneu	AD	1330	33	4	3	C	5	1	0	1	0	3	3	2	3	4	2	2	1	0	1	2	4	1	2	1	2	4	0	0	1	2	1	✔			
2	Limbic	4	81	M	0	0	Lung ca	Metastatic tumor	1240	33	2	1	A	1	1	1	1	1	3	3	2	3	3	3	2	1	0	0	2	2	1	1	1	1	3	0	0	0	1	1	✔			
2	Limbic	5	77	F	0	0	AD, Pneu	AD	962	33	6	3	C	5	1	2	0	0	0	1	2	1	4	2	2	1	0	0	3	3	0	0	0	0	0	0	0	0	0	0	✔			
2	Limbic	5	88	M	0	0	CBS, Pneu	PSP	1248	33	1	1	A	1	1	3	0	0	2	2	2	2	1	2	1	1	2	0	0	2	1	0	2	0	1	0	0	0	0	0	✔			
2	Limbic	4	99	M	0	0	Pneu, DM	Prodromal LBD	1350	33	2	3	C	3	1	1	1	0	2	1	2	2	3	2	1	0	0	0	2	1	0	2	2	1	3	0	0	0	1	1	✔	✔		
2	Limbic	4	82	M	0	0	OMI, DM, CRF	NFT dominant change	1094	33	3	1	A	1	2	0	0	0	3	3	2	3	3	2	1	1	0	1	2	3	0	0	2	1	3	0	0	0	1	1	✔			
2	Limbic	4	93	M	0	0	AD, Pneu	AD & CVD	1307	34	4	2	B	4	0.5	1	0	0	2	1	2	2	3	3	2	1	0	0	2	2	0	0	1	0	2	0	0	0	0	0	✔			
2	Limbic	4	89	M	1	0	Sepsis	CVD	1238	33	2	1	0	1	0.5	1	0	0	3	3	3	3	3	3	3	1	1	1	3	1	0	1	1	1	2	0	0	0	1	1	✔			
2	Diffuse neocortical	5	91	F	0	0	MDD, BI	Prodromal LBD	1040	33	2	1	0	1	2	1	1	1	2	3	2	3	4	4	3	2	2	2	2	3	0	0	1	1	2	0	0	0	1	0	✔			
2	Diffuse neocortical	6	89	M	0	0	Pneu	Prodromal LBD	1144	33	2	3	B	4	1	2	0	0	2	2	2	3	4	4	3	3	2	2	2	2	0	2	0	2	3	0	1	1	2	1	✔			
2	Diffuse neocortical	5	68	M	0	0	Liver cirrhosis	Prodromal LBD	1666	33	2	1	0	1	2	0	1	0	3	3	2	3	3	2	2	2	2	1	NA	NA	1	2	1	2	2	0	0	0	2	0	✔			
2	Limbic	4	83	F	1	1	PSP, Pneu	PSP	1114	33	3	1	0	2	2	0	1	NA	2	2	2	2	3	3	2	1	1	0	2	2	0	0	2	1	3	0	0	1	1	1	✔			
2	Diffuse neocortical	5	70	F	0	0	AML, DM	Prodromal LBD	1276	34	1	1	A	2	1	1	0	0	3	3	2	3	4	2	3	2	2	2	2	2	0	0	0	2	3	0	0	0	2	0	✔			
2	Brainstem-predominant	4	93	F	1	0	Ca of UP	Prodromal LBD	1016	33	3	1	A	5	1	1	1	0	2	2	2	2	2	1	0	0	1	0	2	2	1	0	0	0	1	0	0	0	0	0	✔			
2	Diffuse neocortical	5	81	M	0	0	ML, DM	IVL	1166	33	0	0	0	0	1	0	1	1	2	2	2	3	3	2	2	2	2	1	2	2	1	2	1	0	2	0	0	0	0	0	✔			
2	Diffuse neocortical	5	82	M	0	0	Stomach ca	CVD	1347	33	3	1	0	1	0	1	0	0	3	3	2	3	3	3	3	2	2	2	1	1	0	3	1	2	3	1	1	1	1	2	✔			
2	Limbic	4	98	M	1	0	Ileus	AD change	1203	33	3	2	C	5	1	1	0	0	1	1	2	2	3	2	2	0	0	0	4	2	0	0	0	0	2	0	0	0	0	0	✔			
2	Diffuse neocortical	5	69	M	0	0	Pneu	CVD	935	33	2	1	0	1	1	1	0	0	2	2	2	3	3	2	2	2	1	1	2	1	0	0	1	0	2	0	0	0	0	0	✔			
2	Limbic	4	88	M	1	0	PAF, GI bleed	PAF & CVD	990	33	2	1	B	2	1	1	0	0	3	3	3	3	3	2	2	1	1	1	1	2	1	3	1	2	3	0	0	0	2	1	✔			
3	Diffuse neocortical	6	83	M	1	1	PD (HY5), Pneu	PD	1170	33	3	1	0	1	0.5	1	1	1	3	3	3	3	4	2	4	2	1	0	1	2	0	2	1	2	3	0	1	0	2	1	✔			
3	Diffuse neocortical	6	81	M	1	1	PD (HY3), Pneu	PD	1208	33	2	1	A	1	0.5	1	1	0	3	3	3	3	4	3	3	2	2	2	2	3	1	1	2	2	3	0	0	1	2	1	✔			
3	Diffuse neocortical	5	88	M	0	1	Pneu	PD	1308	23	3	1	A	1	1	1	0	0	1	3	3	3	4	1	2	2	2	0	2	3	1	2	0	2	3	1	1	1	2	1	✔		✔	✔
3	Diffuse neocortical	6	84	F	1	1	PD (HY5), Pneu	PD & PSP	1252	33	2	1	A	1	1	0	0	0	3	3	3	3	4	3	2	2	1	0	2	2	0	2	1	1	3	0	0	0	1	0	✔			
3	Diffuse neocortical	5	76	F	1	1	PD (HY2)	PD	1460	33	2	1	B	1	0.5	0	0	0	2	2	2	3	3	2	2	2	2	2	1	2	0	3	1	2	2	0	0	0	2	1	✔			
3	Brainstem-predominant	4	90	F	0	1	PD (HY3), SAH	PD	1297	33	2	1	0	1	0.5	2	0	1	1	2	3	1	1	1	2	1	0	1	2	2	0	0	0	0	1	0	0	0	0	0	✔			
3	Limbic	4	95	M	0	1	IHD	PD	1263	33	2	1	A	2	2	1	1	1	1	2	2	2	2	2	2	1	1	1	2	2	0	0	1	0	3	0	0	0	0	0	✔			
3	Diffuse neocortical	5	75	M	1	1	PD (HY4), Colon ca	PD	1441	33	3	1	0	2	0.5	3	1	1	3	3	3	3	3	3	3	2	1	1	1	3	0	2	1	2	3	0	0	0	2	2	✔			
4	Diffuse neocortical	6	84	M	1	1	LBD, Prostate ca	PDD/DLBT	1129	23	2	1	A	3	1	3	1	0	3	2	3	3	4	3	3	3	2	2	2	2	1	3	1	2	3	0	0	0	2	1	✔			
4	Diffuse neocortical	6	79	F	1	1	PD (HY4)	PDD/DLBT	1210	NA	2	1	A	1	0.5	1	1	NA	3	3	3	3	4	3	4	2	1	1	2	3	1	2	1	2	3	0	0	0	2	0	✔			
4	Limbic	4	80	F	1	1	PD (HY5), Pneu	PDD/DLBT	1194	23	2	1	A	0	0.5	0	1	0	3	3	3	3	4	3	2	0	1	0	2	4	1	2	1	2	3	1	1	1	2	1	✔		✔	✔
4	Limbic	5	93	F	0	0	RF	AD & CVD	1180	34	4	3	C	5	2	0	0	0	3	3	3	3	4	3	3	1	1	1	2	4	0	0	1	1	1	0	0	0	1	0	✔			
4	Diffuse neocortical	5	75	M	1	0	Int Perf	PDD/DLBT & AD & CVD	1235	34	6	3	C	5	0.5	2	0	0	3	2	2	2	4	4	3	2	1	1	3	4	0	0	2	1	3	0	0	1	1	0	✔			
4	Diffuse neocortical	6	89	M	1	1	PD (HY5), Pneu	PDD/DLBT	1202	33	2	1	A	1	0.5	0	1	0	3	3	3	3	4	2	3	2	2	2	2	3	2	2	2	3	3	0	1	1	3	1	✔		✔	✔
4	Diffuse neocortical	6	87	M	1	1	PD (HY5), Pneu	PDD/DLBT	1271	33	3	1	0	1	1	0	1	0	3	2	3	3	4	3	3	2	2	2	3	4	0	1	1	2	3	0	0	0	2	0	✔			
4	Diffuse neocortical	6	84	F	0	0	BI	PDD/DLBT & AD & CVD	1084	33	4	3	C	3	2	0	0	0	3	3	3	3	4	4	3	3	2	2	2	4	0	0	1	2	2	0	0	0	2	2	✔	✔		✔
4	Diffuse neocortical	5	81	M	1	1	PD (HY5), Pneu	PDD/DLBT	1338	33	2	1	A	1	2	0	0	0	3	3	3	3	4	3	3	2	2	2	1	2	0	2	1	2	3	0	0	2	2	0	✔			
4	Diffuse neocortical	6	91	F	0	1	PDD, Pneu	PDD/DLBT & AGD	1208	33	3	3	B	4	3	2	1	0	2	2	2	3	3	3	3	2	2	2	2	2	0	0	1	1	2	0	0	1	1	0	✔			✔
4	Diffuse neocortical	6	93	F	1	1	DLB, AAneu	PDD/DLBT	1140	34	3	1	B	2	0.5	3	1	1	2	2	2	3	4	2	3	2	2	2	1	3	1	2	1	3	3	0	0	1	3	2	✔			
4	Diffuse neocortical	5	86	F	1	1	PDD, Pneu	PDD/DLBT	1147	33	3	0	0	0	1	2	0	1	3	2	3	3	4	3	3	2	2	2	1	4	1	2	1	2	3	0	1	1	2	1	✔			
4	Diffuse neocortical	5	71	F	0	0	Pneu	PDD/DLBT	1254	34	2	1	0	2	0	2	1	0	3	3	3	3	3	3	2	2	2	2	1	2	0	2	1	2	3	0	0	0	2	1	✔			
4	Diffuse neocortical	5	95	F	0	0	CRF	PDD/DLBT & AD	1198	33	5	3	C	5	1	1	1	0	3	3	2	3	4	3	2	2	1	2	3	3	1	0	1	0	3	0	0	0	0	0	✔			
4	Diffuse neocortical	5	68	M	0	0	CBS, Pneu	PDD/DLBT & AD	1213	33	6	3	C	5	1	1	0	1	2	2	3	3	4	4	3	2	2	2	2	2	0	0	0	0	1	0	0	0	0	0	✔	✔	✔	
4	Diffuse neocortical	6	99	F	0	0	Dementia, Pneu	PDD/DLBT	1088	33	3	1	A	1	1	3	1	2	3	3	3	3	4	3	3	3	2	2	2	1	0	1	1	3	3	0	1	0	3	1	✔			
4	Diffuse neocortical	5	92	M	1	1	DLB, Pneu	PDD/DLBT	1343	33	1	1	A	1	0.5	1	0	0	3	2	3	3	4	3	3	2	2	2	1	2	1	3	1	2	3	0	0	0	2	2	✔			
4	Diffuse neocortical	5	87	F	0	1	DLB, Sepsis	PDD/DLBT & AD	1135	33	5	3	C	5	0.5	2	2	0	2	2	2	2	3	3	3	3	2	1	1	2	0	2	1	1	3	0	0	0	1	1	✔			
4	Diffuse neocortical	6	85	F	0	0	AAD	PDD/DLBT & AGD	1188	34	2	3	B	3	3	3	1	2	3	3	3	3	4	3	3	2	2	2	2	2	1	0	1	1	2	0	0	0	1	1	✔			
5	Diffuse neocortical	6	86	F	0	1	AD	DNTC	1115	34	6	3	C	5	1	3	2	0	3	3	3	3	4	4	4	4	4	4	2	4	0	0	2	2	3	1	0	0	2	1	✔			
5	Diffuse neocortical	5	104	F	0	0	OMI	PDD/DLBN & AD	1045	33	4	3	C	3	2	3	1	0	3	3	3	3	4	4	4	2	3	2	2	4	0	0	1	2	3	1	0	1	2	1	✔			
5	Diffuse neocortical	6	70	F	1	1	DLB, Pneu	PDD/DLBN & AD	1115	34	4	3	C	5	1	1	1	0	3	3	3	3	4	4	4	4	4	3	2	4	0	2	1	1	2	0	0	0	1	1	✔	✔		
5	Diffuse neocortical	6	79	F	0	1	AD, Colon ca	PDD/DLBN & AD & CVD	758	33	6	3	C	5	1	3	0	0	1	2	2	3	4	4	3	4	2	2	2	4	0	0	0	0	1	0	0	0	0	0	✔	✔		
5	Diffuse neocortical	5	96	F	0	0	Pneu	PDD/DLBN & AD	1032	33	4	3	C	4	2	0	0	0	3	3	3	3	4	4	4	3	3	2	2	4	1	2	1	2	4	1	1	1	2	1	✔	✔	✔	✔
5	Diffuse neocortical	5	84	M	1	1	DLB, GI bleed	PDD/DLBN & AGD	1414	33	2	2	A	3	3	1	0	0	3	3	3	3	4	4	4	3	3	2	2	4	0	1	0	2	3	1	1	1	2	1	✔	✔		
5	Diffuse neocortical	6	87	M	1	0	DLB, Pneu	PDD/DLBN	1183	34	2	3	C	5	1	2	0	2	3	3	3	3	4	4	4	3	3	3	2	3	1	2	2	2	3	0	1	1	2	1	✔			✔
5	Diffuse neocortical	6	80	F	0	1	DLB, Ca of UP	PDD/DLBN & CVD	1127	33	2	1	B	3	1	0	0	0	3	3	3	4	4	4	4	3	3	2	1	4	0	1	1	2	3	0	0	0	2	1	✔			
5	Diffuse neocortical	6	88	F	1	0	CHF	PDD/DLBN	1228	33	2	3	C	4	1	1	0	0	3	3	3	3	4	4	4	3	3	3	3	4	0	2	1	2	3	0	0	0	2	1	✔			
5	Diffuse neocortical	6	93	F	1	1	PD (HY5), AMI	PDD/DLBN	958	33	2	1	0	1	1	3	1	1	3	3	3	3	4	4	3	3	3	3	2	4	1	0	1	2	3	0	0	1	2	2	✔			✔
5	Diffuse neocortical	5	99	F	1	1	DLB, Pneu	PDD/DLBN & AD & CVD	1253	33	5	3	C	4	2	3	0	0	3	3	3	3	4	4	4	3	3	3	3	4	0	0	0	1	3	0	0	0	0	1	✔			
5	Limbic	6	82	M	1	1	DLB	PDD/DLBN	1396	33	2	1	0	1	0.5	0	0	0	3	3	2	3	3	2	2	2	1	0	2	2	0	2	1	2	3	0	0	0	2	1	✔			
5	Diffuse neocortical	6	90	F	1	1	DLB, CHF	PDD/DLBN & AD	1281	33	4	3	C	5	1	3	2	0	3	3	3	3	4	4	4	3	3	3	1	2	0	0	1	2	3	0	0	0	2	0	✔			
5	Diffuse neocortical	6	91	F	0	0	Pneu	PDD/DLBN & AD	1004	44	5	3	C	5	1	3	1	0	2	3	3	3	4	4	3	3	3	2	2	3	0	0	1	3	3	0	1	1	3	1	✔		✔	✔
5	Diffuse neocortical	5	83	M	0	1	DLB, Pneu	PDD/DLBN	1227	33	1	1	B	3	2	1	0	0	2	2	2	3	4	3	3	2	2	2	2	2	0	3	1	2	3	0	0	1	2	1	✔			
5	Diffuse neocortical	5	86	M	1	1	PD (HY5)	PDD/DLBN	1196	23	3	0	0	1	1	3	1	1	3	2	3	3	3	3	3	2	2	1	2	3	0	1	1	2	3	0	0	0	2	1	✔			
5	Diffuse neocortical	5	86	M	0	1	AD, DLB, Pneu	PDD/DLBN & AD & CVD	1267	44	5	3	C	5	2	3	0	1	3	3	3	3	4	4	3	3	2	2	3	3	0	0	1	1	3	0	0	1	1	1	✔			
5	Diffuse neocortical	6	85	F	1	1	PD (HY5), Pneu	PDD/DLBN	1106	33	2	3	B	5	0	1	1	1	3	3	3	3	4	4	4	3	3	3	1	4	1	2	1	2	3	1	1	1	2	2	✔			
5	Diffuse neocortical	6	83	F	0	0	AD, Pneu	PDD/DLBN & AD	1225	34	4	3	B	5	2	3	1	0	3	3	3	3	4	4	4	4	4	4	3	4	1	2	1	2	3	0	1	1	2	1	✔			

Supplementary Table 1 Cause of death for the 518 BBAR cases at autopsy

	Number of the cases (%)		With neurodegenerative disorder		Without neurodegenerative disorder	
**Respiratory disease**	**140**	**(27.0)**	**64**	**(12.4)**	**76**	**(14.7)**
Pneumonia	96	(18.5)	46	(8.9)	50	(9.7)
Organizing pneumonia	20	(3.9)	13	(2.5)	7	(1.4)
Interstitial pneumonia	11	(2.1)	1	(0.2)	10	(1.9)
Diffuse alveolar damage	6	(1.2)	2	(0.4)	4	(0.8)
Emphysema	5	(1.0)	1	(0.2)	4	(0.8)
Pulmonary edema	1	(0.2)	1	(0.2)	0	(0)
Pneumoconiosis	1	(0.2)	0	(0)	1	(0.2)
**Malignant neoplasm**	**138**	**(26.6)**	**21**	**(4.1)**	**117**	**(22.6)**
Lung	32	(6.2)	8	(1.5)	24	(4.6)
Leukemia	15	(2.9)	0	(0)	15	(2.9)
Lymphoma	14	(2.7)	0	(0)	14	(2.7)
Stomach	14	(2.7)	3	(0.6)	11	(2.1)
Large intestine	11	(2.1)	4	(0.8)	7	(1.4)
Brain	10	(1.9)	0	(0)	10	(1.9)
Pancreas	7	(1.4)	1	(0.2)	6	(1.2)
Prostate gland	5	(1.0)	1	(0.2)	4	(0.8)
Gallbladder	4	(0.8)	1	(0.2)	3	(0.6)
Urinary bladder	4	(0.8)	1	(0.2)	3	(0.6)
Others	18	(3.5)	2	(0.4)	16	(3.1)
**Cardiovascular disease**	**100**	**(19.3)**	**18**	**(3.5)**	**82**	**(15.8)**
Acute myocardial infarction	31	(6.0)	7	(1.4)	24	(4.6)
Acute aortic dissection	14	(2.7)	2	(0.4)	12	(2.3)
Aortic aneurysm	10	(1.9)	2	(0.4)	8	(1.5)
Aortic valve stenosis	8	(1.5)	1	(0.2)	7	(1.4)
Chronic myocardial infarction	8	(1.5)	1	(0.2)	7	(1.4)
Pulmonary embolism	5	(1.0)	1	(0.2)	4	(0.8)
Dilated cardiomyopathy	4	(0.8)	1	(0.2)	3	(0.6)
Infective endocarditis	4	(0.8)	0	(0)	4	(0.8)
Others	16	(3.1)	3	(0.6)	13	(2.5)
**Cerebrovascular disease**	**33**	**(6.4)**	**3**	**(0.6)**	**30**	**(5.8)**
Infarction	19	(3.7)	1	(0.2)	18	(3.5)

Hemorrhage	9	(1.7)	1	(0.2)	8	(1.5)
Subarachnoid hemorrhage	4	(0.8)	1	(0.2)	3	(0.6)
Subdural hemorrhage	1	(0.2)	0	(0)	1	(0.2)
**Digestive disease**	**33**	**(6.4)**	**13**	**(2.5)**	**20**	**(3.9)**
Ischemic enteritis	8	(1.5)	4	(0.8)	4	(0.8)
Intestinal perforation	5	(1.0)	5	(1.0)	0	(0)
Duodenal ulcer	4	(0.8)	0	(0)	4	(0.8)
Liver cirrhosis	3	(0.6)	0	(0)	3	(0.6)
Others	13	(2.5)	4	(0.8)	9	(1.7)
**Systemic infectious disease**	**25**	**(4.8)**	**10**	**(1.9)**	**15**	**(2.9)**
Sepsis	13	(2.5)	3	(0.6)	10	(1.9)
Tuberculosis	6	(1.2)	5	(1.0)	1	(0.2)
Nontuberculous mycobacteria infection	2	(0.4)	0	(0)	2	(0.4)
Others	4	(0.8)	2	(0.4)	2	(0.4)
**Nervous system disease**	**16**	**(3.1)**	**12**	**(2.3)**	**4**	**(0.8)**
Amyotrophic lateral sclerosis	7	(1.4)	7	(1.4)	0	(0)
Frontotemporal lobar degeneration	2	(0.4)	2	(0.4)	0	(0)
Others	7	(1.4)	3	(0.6)	4	(0.8)
**Connective tissue disease**	**12**	**(2.3)**	**1**	**(0.2)**	**11**	**(2.1)**
Rheumatoid arthritis	4	(0.8)	1	(0.2)	3	(0.6)
Spondylodiscitis	3	(0.6)	0	(0)	3	(0.6)
Others	5	(1.0)	0	(0)	5	(1.0)
**Metabolic disease**	**8**	**(1.5)**	**3**	**(0.6)**	**5**	**(1.0)**
Senile systemic amyloidosis	4	(0.8)	3	(0.6)	1	(0.2)
Amyloid light-chain amyloidosis	3	(0.6)	0	(0)	3	(0.6)
Pellagra	1	(0.2)	0	(0)	1	(0.2)
**Injury and external causes**	**6**	**(1.2)**	**4**	**(0.8)**	**2**	**(0.4)**
Tracheobronchial foreign body	5	(1.0)	4	(0.8)	1	(0.2)
Ruptured spleen	1	(0.2)	0	(0)	1	(0.2)
**Genitourinary disease**	**5**	**(1.0)**	**4**	**(0.8)**	**1**	**(0.2)**
Chronic kidney disease	3	(0.6)	2	(0.4)	1	(0.2)
Pyelonephritis	2	(0.4)	0	(0)	2	(0.4)
**Hematopoietic disease**	**2**	**(0.4)**	**0**	**(0)**	**2**	**(0.4)**
**Total**	**518**	**(100)**	**153 (29.5)**		**365 (70.5)**	

BBAR, the Brain Bank for Aging Research.

Supplementary Table 2 Incidence of Lewy pathology in the PNS

BBAR LB stage	Number of cases (Mean Age at death)		Esophagus *n* (%)		Sympathetic ganglia *n* (%)		Heart *n* (%)		Adrenal gland *n* (%)		Skin *n* (%)	
0.5	29	(81.2, SD 9.6)	1	(3.4)	9	(31.0)	3	(10.3)	1	(3.4)	1	(3.4)
1	83	(82.9, SD 10.1)	23	(27.7)	51	(61.4)	38	(45.8)	19	(22.9)	8	(9.6)
2	20	(83.8, SD 9.5)	13	(65.0)	19	(95.0)	15	(75.0)	10	(50.0)	7	(35.0)
3	8	(84.0, SD 6.8)	6	(75.0)	8	(100)	6	(75.0)	6	(75.0)	2	(25.0)
4	19	(85.2, SD 8.2)	17	(89.5)	19	(100)	18	(94.7)	12	(63.2)	9	(47.4)
5	19	(86.9, SD 7.6)	18	(94.7)	19	(100)	16	(84.2)	11	(57.9)	5	(26.3)
Total	178		78	(43.8)	125	(70.2)	98	(55.1)	60	(33.7)	32	(18.0)

BBAR, the Brain Bank for Aging Research; *LB*, Lewy body; *SD*, standard deviation.

Supplementary Table 3 Summary of 9 cases with PNS-only Lewy pathology

Age	Gender	AF	PA	NPD	BW	NFT	SP	BBAR LB	Lewy pathology*															
									DMNV	LC	SN	nbM	Amy	TrE	Cing	T	F	P	OB	Skin	Adr	He	Eso	SY
74	M	0	0	CVD	1318	1	1	0.5	0	0	0	0	0	0	0	0	0	0	0	0	0	0	0	1
85	F	0	0	CVD	1183	2	1	0.5	0	0	0	0	0	0	0	0	0	0	0	0	0	0	0	1
96	F	0	0	NFTD CVD	1032	3	1	0.5	0	0	0	0	0	0	0	0	0	0	0	0	0	0	0	1
49	M	0	0	CVD	1466	1	0	1	0	0	0	0	0	0	0	0	0	0	0	0	0	0	0	3
71	M	0	0	Pr LBD	1507	1	1	1	0	0	0	0	0	0	0	0	0	0	0	0	0	2	0	0
77	F	0	0	Pr LBD	1252	2	0	1	0	0	0	0	0	0	0	0	0	0	0	0	0	1	0	3
83	F	0	0	AGD	1123	3	1	1	0	0	0	0	0	0	0	0	0	0	0	0	0	2	0	1
89	M	0	0	Cont	1122	2	2	1	0	0	0	0	0	0	0	0	0	0	0	0	0	2	0	2
93	M	1†	0	SDH	1122	3	3	1	0	0	0	0	0	0	0	0	0	0	0	0	0	0	0	2

Adr, adrenal gland; *AF*, autonomic failure. 1 = presence, 0 = absence; *AGD*, argyrophilic grain disease; *Amy*, amygdala; *BBAR LB*, Lewy body stage of the Brain Bank for Aging Research; *BW*, brain weight (g); *Cont*, contusion; *CVD*, cerebrovascular disease; *DLB*, dementia with Lewy bodies; *DMNV*, dorsal motor nucleus of the vagus; *Eso*, esophagus; *F*, frontal lobe; *He*, heart; *LBD*, Lewy body disease; *LC*, locus coeruleus; *M*, medullar oblongata; *nbM*, nucleus basalis of Meynert; *NFT*, Braak neurofibrillary tangle stage; *NFTD*, neurofibrillary tangle predominant dementia; *NPD*, neuropathological diagnosis; *OB*, olfactory bulb; *P*, parietal lobe; *PA*, Parkinsonism. 1 = presence, 0 = absence; *Pr LBD*, preclinical LBD; *SDH*, subdural hemorrhage; *SN*, substantia nigra; *SP*, Braak senile plaque stage; *SY*, sympathetic ganglia; *T*, temporal lobe; *TrE*, transentorhinal cortex.

*Semi-quantitative analyses of the α-synuclein deposition (score 0–4 according to DLB Consensus guidelines).

†Constipation.

Supplementary Table 4 Summary of the literature on Lewy pathology in the ENS*

Author	Year	Region	Number of subjects	Positivity of Lewy pathology				
				PD	DLB	PAF	Preclinical/ Prodromal LBD	Control
**Autopsy**								
Tanei Z	2020	L-Eso	518	6/8 (75%)	35/38 (92%)	1/1	Earliest LBD: 1/29 (3%) Prec/Prod LBD: 35/102 (34%)	0/340 (0%)
Beach TG	2016	S-Col	10	5/5 (100%)				0/5 (0%)
Gelpi E	2014	L-Eso, Sto Ile, T/D-Col Rec	28	8/10 (80%)	5/5 (100%)			1/13 (8%)
Mu L	2013	Pharynx	14	10/10 (100%)				0/4 (0%)
Gold A	2013	Col	95	10/10 (100%)				Gen 40/77 (52%) AD 3/8 (38%)
Annerino DM	2012	Sto, Duo, Ile, T-Col, Rec	29	13/13 (100%)			2/4 (50%)	0/12 (0%)
Del Tredici K	2011	L-Eso, Sto	4	3/3 (100%)			1/1 (100%)	
Beach TG	2010	U/L-Eso, Sto Duo, Jej, Ile Rec, T/S-Col Liv, Pan, Gal	92	11/17 (65%)	5/9 (56%)		1/7 (14%)	

Braak H	2006	L-Eso, Sto	10	3/3 (100%)	2/2 (100%)	0/5 (0%)
Bloch A	2006	L-Eso	98	1/2 (50%)	14/17 (82%)	
Wakabayashi K†	1988	U/M/L-Eso	31	Eso 8/8 (100%)	Eso 8/24 (33%)	
		Sto, Duo		Sto 2/8 (25%)	Sto 0/24 (0%)	
		Jej, Ile		Duo 1/7 (14%)	Duo 3/24 (13%)	
		A/T/D-Col		Jej 2/8 (25%)	Jej 1/24 (4%)	
		Rec		Ile 0/8 (0%)	Ile 1/24 (4%)	
				Col 3/8 (38%)	Col 0/24 (0%)	
				Rec 2/7 (29%)	Rec 0/24 (0%)	
Qualman SJ†	1984	L-Eso, Sto	80	2/22 (9%)		0/50 (0%)
		Jej, Ile, Col				
		Rec				
**Surgery**						
Yan, F.	2018	Sto, Col	63	Sto 6/10 (60%)		Sto 1/11 (9%)
		Rec, App		Col 5/9 (56%)		Col 2/9 (22%)
				Rec 5/9 (56%)		Rec 2/8 (25%)
				App 1/3 (33%)		App 2/8 (25%)
Shin C‡	2017	Sto	66	Sto 7/12 (58%)		
		A/T/D-Col		A/T-Col 3/9 (33%)		
		Rec		D-Col/Rec 2/12 (17%)		
Kim JS	2017	Col	12			10/12 (83%)
Stokholm MG‡	2016	Eso, Sto	147	Eso 4/7 (57%)	Eso 2/16 (13%)	Eso 2/23 (9%)
		SmI, App		Sto 4/10 (40%)	Sto 9/21 (43%)	Sto 5/31 (16%)

		Col		SmI 1/1 (100%)		SmI 6/7 (86%)	SmI 3/8 (38%)
				App 2/3 (67%)		App 5/5 (100%)	App 6/8 (75%)
				Col 3/5 (60%)		Col 4/8 (50%)	Col 5/13 (38%)
Aldecoa I	2015	Sto, SmI, Col	18	4/6 (67%)			1/12 (8%)
Kurashiki-Osaka T	2014	Sto	1		1/1		
Masuda H	2014	T-Col	1		1/1		
Ito S	2014	Sto, SmI	8	4/6 (67%)		2/2 (100%)	
		Col, Gal					
Sunwoo MK	2013	Sto	32	8/32 (25%)			
Bottner M	2012	Col, Rec	13				11/13 (85%)
Minguez-Castellanos	2007	Sto, SmI	77				3/77 (4%)
A		LaI					
**Biopsy**							
Chahine LM	2020	S-Col	80	8/59 (14%)			21/21 (100%)
Leclair-Visonneau L	2019	S-Col	43	20/43 (47%)			
	2017						
Fenyi A	2019	S-Col, Rec	29	S-Col 5/10 (50%)			S-Col 0/1 (0%)
				Rec 0/4 (0%)			Rec 0/2 (0%)
Lee HJ	2018	Sto, A/T/S-Col	77	12/35 (34%)			20/52 (38%)
Barrenschee M	2017	Rec	23	3/12 (25%)			4/11 (36%)
Chung SJ	2016	Sto	104	Sto 4/11 (36%)			Sto 10/19 (53%)
		A/T/S-Col		A-Col 2/25 (8%)			A-Col 5/25 (20%)
				T-Col 5/24 (21%)			T-Col 8/36 (22%)

				S-Col 0/18 (0%)		S-Col 2/20 (10%)
Visanji NP	2015	S-Col, Rec	33	22/22 (100%)		9/11 (91%)
Sprenger FS	2015	A/T/D/S-Col	50	1/19 (5%)	4/17 (24%)	0/14 (0%)
Sanchez-Ferro A	2015	Sto	57	17/28 (61%)	1/6 (17%)	1/23 (4%)
Hilton D	2014	Eso, Sto, Gal<br>SmI, LaI	223	7/62 (11%)		0/161 (0%)
Devos D	2013	A-Col	18	12/18 (67%)		
Shannon KM	2012	S-Col	55	9/9 (100%)		Gen 0/23 (0%)<br>IBD 0/23 (0%)
Shannon KM	2012	Col	3		3/3 (100%)	
Pouclet H	2012	A/D-Col<br>Rec	35	A-Col 17/26 (65%)<br>D-Col 11/26 (42%)<br>Rec 6/26 (23%)		0/9 (0%)
Pouclet H	2012	D/S-Col	19	5/9 (56%)		0/10 (0%)
Lebouvier T	2010	A/D-Col	39	21/29 (72%)		0/10 (0%)
Lebouvier T	2008	A-Col	10	4/5 (80%)		0/5 (0%)

AD, Alzheimer's disease; *App*, Appendix; *Col*, colon; *D-col*, Descending colon; *Duo*, Duodenum; *Gal*, Gallbladder; *Gen*, General Population; *IBD*, Inflammatory bowel disease; *Ile*, Ileum; *Jej*, Jejunum; *LaI*, Large intestine; *L-Eso*, Lower esophagus; *Liv*, Liver; *M-Eso*, Middle esophagus; *Pan*, Pancreas; *Prec/Prod*, Preclinical/Prodromal; *Rec*, Rectum; *SmI*, Small intestine; *Sto*, Stomach; *S-Col*, Sigmoid colon; *T-Col*, Transverse colon; *U-Eso*, Upper esophgagus.

*Ref. Wakabayashi K Wakabayashi K, Miki Y (2018) [Multi-Organ Distribution of Alpha-Synuclein Pathology in Dementia with Lewy Bodies]. Brain Nerve 70: 489-500 Doi 10.11477/mf.1416201031

†These studies examined the occurrence of LB before the development of phosphorylated α-synuclein immunohistochemistry.

‡Surgical and biopsy specimens.
